# The unique evolution of the programmed cell death 4 protein in plants

**DOI:** 10.1186/1471-2148-13-199

**Published:** 2013-09-16

**Authors:** Shijun Cheng, Renyi Liu, Daniel R Gallie

**Affiliations:** 1 Department of Biochemistry, University of California, Riverside, CA 92521-0129, USA; 2 Department of Botany and Plant Sciences, University of California, Riverside, CA 92521-0129, USA

**Keywords:** MA3 domain, PDCD4, eIF4G, eIF4A, Translation initiation, Domain duplication, Hormone signaling

## Abstract

**Background:**

The programmed cell death 4 (PDCD4) protein is induced in animals during apoptosis and functions to inhibit translation and tumor promoter-induced neoplastic transformation. PDCD4 is composed of two MA3 domains that share similarity with the single MA3 domain present in the eukaryotic translation initiation factor (eIF) 4G, which serves as a scaffold protein to assemble several initiation factors needed for the recruitment of the 40S ribosomal subunit to an mRNA. Although eIF4A is an ATP-dependent RNA helicase that binds the MA3 domain of eIF4G to promote translation initiation, binding of eIF4A to the MA3 domains of PDCD4 inhibits protein synthesis. Genes encoding PDCD4 are present in many lower eukaryotes and in plants, but PDCD4 in higher plants is unique in that it contains four MA3 domains and has been implicated in ethylene signaling and abiotic stress responses. Here, we examine the evolution of PDCD4 in plants.

**Results:**

In older algal lineages, PDCD4 contains two MA3 domains similar to the homolog in animals. By the appearance of early land plants, however, PDCD4 is composed of four MA3 domains which likely is the result of a duplication of the two MA3 domain form of the protein. Evidence from fresh water algae, from which land plants evolved, suggests that the duplication event occurred prior to the colonization of land. PDCD4 in more recently evolved chlorophytes also contains four MA3 domains but this may have resulted from an independent duplication event. Expansion and divergence of the PDCD4 gene family occurred during land plant evolution with the appearance of a distinct gene member following the evolution of basal angiosperms.

**Conclusions:**

The appearance of a unique form of PDCD4 in plants correlates with the appearance of components of the ethylene signaling pathway, suggesting that it may represent the adaptation of an existing protein involved in programmed cell death to one that functions in abiotic stress responses through hormone signaling.

## Background

Following transcription and processing of an mRNA, the ribosome is responsible for performing protein synthesis. Although the bacterial 30 S ribosomal subunit can identify the initiation codon through base-pairing between the 3′-end of its16 S ribosomal RNA subunit and the Shine-Dalgarno sequence upstream of the initiation codon, the 40 S ribosomal subunit of the eukaryotic 80 S ribosome requires several translation initiation factors (eIFs) for its binding to an mRNA and to identify the initiation codon [1-3]. eIF4F, which is composed of eIF4E, eIF4A, and eIF4G, is required to promote 40 S subunit binding to an mRNA. While eIF4E binds to the 5′-cap structure, eIF4A is an ATP-dependent RNA helicase that hydrolyzes ATP in order to unwind secondary structure present in the 5′-leader of an mRNA that would otherwise inhibit 40S subunit scanning during its search for the initiation codon [2]. The helicase activity of eIF4A is enhanced by eIF4B which interacts directly with eIF4A [4-6]. eIF4G is a scaffolding protein that interacts with eIF4E, eIF4A, eIF4B, eIF3 (also required for 40S binding to an mRNA), and the poly(A) binding protein (PABP) [2,4,7-9]. The interaction of eIF4G with eIF4E bound to the 5′-cap and PABP bound to the poly(A) tail circularizes an mRNA and stimulates translation by promoting 40 S subunit recruitment [10,11].

eIF4A binds to two regions of eIF4G that fold into HEAT (Huntington, Elongation Factor 3, PR65/A, TOR) domains, characterized by the presence of antiparallel α-helical hairpins known as HEAT repeats [12,13]. One HEAT domain to which eIF4A binds is present in the middle region of eIF4G (HEAT-1/MIF4G, MIG) which is required for translation while the second is C-distal (eIF4G-MA3, HEAT2/MA3) and serves a regulatory function [14-16]. A third, functionally distinct HEAT domain, present in animal eIF4G but absent from plant eIF4G, does not bind eIF4A but does bind Mnk kinase which phosphorylates eIF4E during active translation [17]. eIF4B and PABP also bind to a site within the central region of eIF4G that partially overlaps the HEAT-1/eIF4G-MIG but they do not bind the HEAT-2/eIF4G-MA3 domain [9], demonstrating the functional diversity of the HEAT-1/eIF4G-MIG and HEAT-2/eIF4G-MA3 domains in their interactions with partner proteins. In addition to eIF4G, plants express an eIF4F isoform called eIFiso4F, which is composed of eIFiso4E, eIF4A, and eIFiso4G [18]. The eIF4B and PABP interaction sites overlap with the eIF4A binding site in the HEAT-1/eIF4G-MIG domain of eIFiso4G more extensively than in eIF4G [19]. As a consequence, eIF4B and PABP compete with eIF4A for binding to the HEAT-1/eIF4G-MIG domain of eIFiso4G in the absence of the HEAT-2/eIF4G-MA3 domain but not in its presence [19].

In addition to eIF4G, other proteins containing an MA3 domain have been described. The programmed cell death 4 (PDCD4) protein is characterized by the presence of two tandem MA3 domains that fold into a subtype of HEAT domains. The N- and C-terminal MA3 domains of human PDCD4 contain four and three helical hairpins, respectively, while eIF4G-MA3 contains five helical hairpins [20]. From a basal level, PDCD4 expression is induced upon programmed cell death in several cell types in mice, including lymphoid and neuronal cells [21]. Overexpressing PDCD4 was unable to induce programmed cell death, suggesting no casual relationship between PDCD4 and apoptosis [21]. Increasing PDCD4 expression, however, was sufficient to inhibit tumor promoter-induced neoplastic transformation while reducing PDCD4 expression resulted in a transformation-sensitive phenotype and the promotion of tumor invasion [22-24]. Animal PDCD4 binds eIF4A which inhibits the latter from binding to eIF4G-MA3 [25-27]. PDCD4 inhibits eIF4A RNA binding and helicase activity and inhibits translation *in vivo*[20,26,28]. PDCD4 binding to eIF4A also disrupts the ATP-binding site and prevents its conformational transition from a nonproductive open state to a productive closed state [28]. PDCD4 binding to eIF4A is required for its ability to inhibit translation and transformation as disruption of eIF4A binding to PDCD4 abolishes its effect on both [26]. The PDCD4 C-terminal MA3 domain contacts the N-terminal domain of eIF4A using structural features conserved with eIF4G-MA3 thereby preventing the interaction between eIF4A and eIF4G-MA3 and inhibiting translation initiation [29,30]. The two MA3 domains of PDCD4 have similar secondary and tertiary structures and either can compete with eIF4A in binding eIF4G-MA3 [20]. Although both MA3 domains of PDCD4 are structurally similar as are their eIF4A-binding surfaces, the two domains function synergistically to bind eIF4A, resulting in a more stable complex with eIF4A [20]. A single PDCD4 MA3 domain is sufficient to inhibit translation but both domains are required to compete effectively with eIF4G-MA3 for binding to eIF4A [28]. PDCD4 also binds eIF4G-MIG without affecting eIF4A binding as they bind to diametrically opposite sides of eIF4A [28]. Binding of PDCD4 to eIF4G-MIG, however, may anchor the binding of eIF4A to eIF4G-MIG thereby preventing its cycling through eIF4G as part of its function during translation initiation [26].

PDCD4 homologs are present in lower eukaryotes and plants although no PDCD4 homolog has been reported in yeast. PDCD4 proteins in higher plants are unique in that they contain four tandem MA3 domains. Higher plants also appear to lack a two MA3 domain PDCD4 homolog. One such four MA3 domain PDCD4 protein (ECIP1) was reported to bind *Arabidopsis thaliana* ethylene receptors, ETR2 and EIN4, as well as EIN2, a downstream component of the ethylene signaling pathway required for the induction of ethylene responses [31]. Loss of ECIP1 expression resulted in increased ethylene sensitivity and tolerance to salt [31]. Thus, like the HEAT-2/ eIF4G-MA3 domain of eIF4G and eIFiso4G, the MA3 domains of ECIP1 are involved in protein-protein interactions. When the PDCD4 homolog containing four MA3 domains first appeared during plant evolution has not been examined. In this study, we have examined the evolution of plant PDCD4-like proteins from a two MA3 domain form in prasinophytes to the appearance of a four MA3 domain form in charophytes and land plants and the likely independent appearance of a four MA3 domain form in Chlamydomonadales. We also examine the expansion and divergence of the PDCD4-like gene family during plant evolution and identify how one distinct gene family member appeared following the evolution of basal angiosperms. How the expansion and divergence of the plant PDCD4 gene family might relate to their function as regulators of ethylene responses is also discussed.

## Results

### MA3-domains are present in multiple proteins in plants

**Figure 1:**
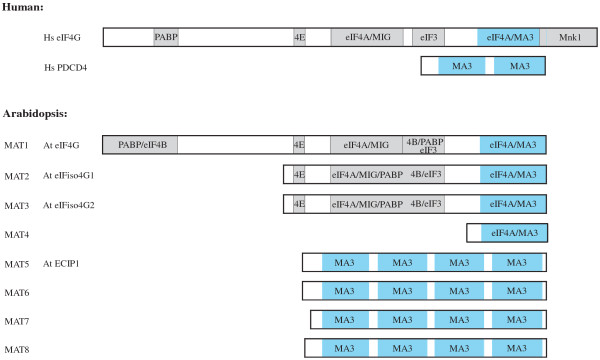
**Domain organization of MA3 domain-containing proteins in *Homo sapiens *and *Arabidopsis thaliana*.** The domain organization of eIF4G and PDCD4 proteins is shown for *H. sapiens* (Hs) and *A. thaliana* (At). For eIF4G, the interaction domains for the translation initiation factors eIF4E, eIF4A, eIF4B, eIF3, as well as the poly(A)-binding protein (PABP) and Mnk kinase are shown. The MA3 domains are shown in blue for eIF4G and for the animal and plant PDCD4 proteins. MAT5-8 comprise the PDCD4 gene family in *A. thaliana*. eIF4G (MAT1, At3g60240), eIFiso4G1 (MAT2, At5g57870), eIFiso4G2 (MAT3, At2g24050), MAT4 (At4g30680), MAT5 (At4g24800), MAT6 (At5g63190), MAT7 (At3g48390), MAT8 (At1g22730).

eIF4G contains a single MA3 domain which in plants, is present at the C-terminus as plant eIF4G lacks the HEAT-3/Mnk-binding domain found in animal eIF4G (Figure 1). Plants express two eIF4G isoforms known as eIF4G and eIFiso4G [18]. Although eIFiso4G is substantially smaller than eIF4G, this is largely accounted for by a shorter N-terminal region [32]. Consequently, eIF4G and eIFiso4G both contain a C-terminal MA3 domain. In Arabidopsis, eIF4G is encoded by a single gene (i.e., At3g60240) whereas eIFiso4G is encoded by two genes, eIFiso4G1 and eIFiso4G2 (i.e., At5g57870 and At2g24050, respectively) [33]. Another gene (At4g30680) encodes a protein composed largely of a MA3 domain that exhibits similarity to the MA3 domains of eIFiso4G1 and eIFiso4G2 (Figure 1).

**Figure 2:**
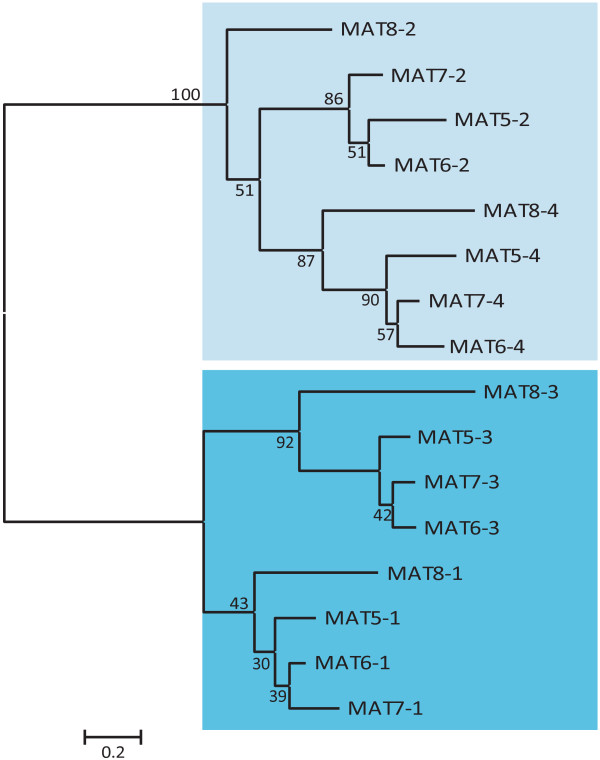
**Phylogenetic analysis of the MA3 domains of *A. thaliana *PDCD4 proteins suggests domain duplication.** A phylogenetic tree of the MA3 domains of *A. thaliana* MAT5-8 proteins was constructed using the maximum-likelihood method. Numbers on each branch denote percentages of bootstrap support. MA3 domains 2 and 4 of each MAT protein are shaded light blue whereas MA3 domains 1 and 3 are shaded dark blue.

In addition to eIF4G, animals express PDCD4, which contains two tandem MA3 domains [21]. A search of higher plants for a similar protein containing two tandem MA3 domains failed to identify a plant homolog. However, a search for other MAT (i.e., MA three) proteins identified four genes, i.e., MAT5 through MAT8, that encode proteins containing four tandem MA3 domains (Figure 1). MAT5 was previously described as ECIP1 which is involved in ethylene signaling and stress tolerance in *Arabidopsis thaliana*[31]. These authors concluded that three such genes were present in the *A. thaliana* genome but the *A. thaliana* gene family actually contains four members: MAT5 (At4g24800), MAT6 (At5g63190), MAT7 (At3g48390), and MAT8 (At1g22730). Comparison of the MA3 domains from MAT5-8 with eIF4G and eIFiso4G revealed conservation of several residues among all MA3 domains but only four residues were invariant in all MA3 domain proteins (Additional file 1). In addition to the invariant residues, the MA3 domains of MAT5-8 exhibited conservation at positions that were, in several instances, distinct from residues conserved among eIF4G and eIFiso4G proteins (Additional file 1), suggesting divergence of MA3 domains in MAT5-8 from those in eIF4G and eIFiso4G. Comparison of the MA3 domains of MAT5-8 suggests that MA3 domains 1 and 3 are distinct from MA3 domains 2 and 4, suggesting duplication of an ancestral two MA3 domain protein. That no gene encoding a two MA3 domain homolog is present in higher plants supports the possibility of duplication. Sequence analysis of the individual MA3 domains of MAT5-8 supported this notion as MA3 domains 1 and 3 are more related to one another as are MA3 domains 2 and 4 (Figure 2). Each MA3 domain from MAT8 was the most divergent from the corresponding MA3 domain of MAT5, MAT6, and MAT7, suggesting that the PDCD4 gene family expanded during plant evolution and diverged into a MAT8 subgroup and a MAT5/6/7 subgroup (see below).

### The four MA3 domain PDCD4 protein is unique to plants

**Figure 3:**
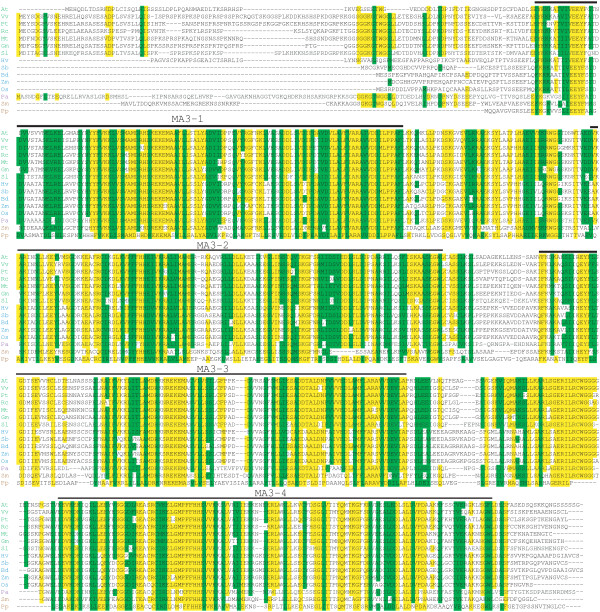
**Sequence alignment of plant PDCD4 homologs.** Alignment of PDCD4 amino acid sequence is shown with amino acid identity relative to *A. thaliana* MAT8 highlighted in yellow amino acid and amino acid similarity highlighted in green. Each MA3 domain present in all proteins is indicated above the pertinent sequence. Protein sequences used were: At, *Arabidopsis thaliana* GenBank: NP_173687 (MAT8); Vv, *Vitis vinifera* GenBank: XP_002264439; Pt, *Populus trichocarpa* GenBank: XP_002307530; Rc, *Ricinus communis* GenBank: XP_002527108; Mt, *Medicago truncatula* GenBank: XP_003619027; Gm, *Glycine max* GenBank: XP_003548962; Sl, *Solanum lycopersicum* GenBank XP_004236309; Hv, *Hordeum vulgare* GenBank: BAK08048; Sb, *Sorghum bicolor* GenBank: XP_002465627; Bd, *Brachypodium distachyon* GenBank: XP_003561877; Zm, *Zea mays* GenBank: NP_001159302; Os, *Oryza sativa* GenBank: AAN05329; Pa, *Picea abies* MA_2314g0010; Sm, *Selaginella moellendorffii* GenBank: XP_002979104; Pp, *Physcomitrella patens* GenBank: XP_001754785.

PDCD4 homologs are present throughout angiosperm species and the proteins exhibit considerable conservation both in domain organization and primary sequence (Figure 3). All higher plant PDCD4 proteins contain four tandem MA3 domains in which MA3 domains 1 and 2 and MA3 domains 3 and 4 are separated by just over 50 residues whereas MA3 domains 1–2 are separated from MA3 domains 3–4 by just over half that length. The N-terminus of plant PDCD4 proteins is highly variable in length and sequence with no conservation across angiosperms (Figure 3). Four MA3 domain PDCD4 homologs are present in the bryophyte *Physcomitrella patens* and in the lycophyte *Selaginella moellendorffii* (Figure 3), demonstrating that the four MA3 domain form of the protein had appeared prior to the evolution of seed plants. A PDCD4 homolog is also present in the gymnosperm species *Picea abies* which is conserved with other plant PCDC4 homologs (Figure 3). A partial *Pinus taeda* cDNA [GenBank: DT628261] exhibited high similarity to MA3 domains 3 through 4 of higher plants (data not shown) suggesting that this gymnosperm species also expresses a four MA3 domain PDCD4 homolog. In contrast, only PDCD4 homologs containing two MA3 domains were found in extensive database searches of animal and lower, non-plant eukaryotic species, indicating that the four MA3 domain PDCD4 homolog is unique to plants.

### The four MA3 domain PDCD4 protein arose during algal evolution

PDCD4 in animals and many lower eukaryotes, including several algal species, contains two MA3 domains while the PDCD4 homolog in other algal species and land plants contains four MA3 domains. This raises the question of when duplication of the two MA3 domain protein into a four MA3 domain protein may have occurred. Examination of the available genome sequences for algae and algal relatives revealed the presence of four MA3 domain PDCD4 homologs in more recently evolved green algae such as *Chlamydomonas reinhardtii* and *Volvox carteri* representing the Chlamydomonadales but only two MA3 domain PDCD4 homologs in algal species of the Mamiellales such as *Micromonas* and *Ostreococcus* (Additional file 2). Only two MA3 domain PDCD4 homologs were observed in heterokonts such as the brown alga *Ectocarpus siliculosus* and in algal relatives such as *Aureococcus anophagefferens*, *Phaeodactylum tricornutum*, *Thalassiisira pseudoonana*, *Phytophthora* species, and *Albugo laibachii* (Additional file 2).

Phylogenetic analysis of MA3 domains 1–2 and MA3 domains 3–4 of *C. reinhardtii* and *V. carteri* PDCD4 with the two MA3 domain PDCD4 homologs from other algae and algal relatives revealed that *C. reinhardtii* and *V. carteri* MA3 domains 1–2 were more similar to the two MA3 domain PDCD4 homologs of prasinophytes or heterokonts than were MA3 domains 3–4 (Additional file 3). MA3 domains 1–2 and MA3 domain 3 of a *Chlorella variabilis* partial PDCD4 protein sequence also clustered with the respective MA3 domains of *C. reinhardtii* and *V. carteri* PDCD4 (Additional file 3). These data suggest that the four MA3 domain PDCD4 homolog in more recently evolved chlorophytes arose from the duplication of a more ancestral, two MA3 domain protein and that, following duplication, MA3 domains 3–4 diverged to a greater extent than did MA3 domains 1–2.

**Figure 4:**
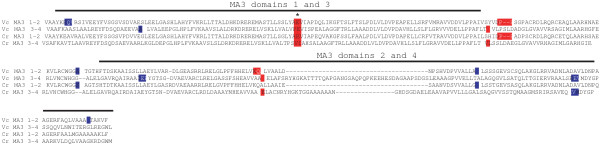
**Intron positions in the *C. reinhardtii *and *V. carteri *PDCD4 genes support domain duplication in the Chlamydomonadales.** Alignment of the sequence comprising MA3 domains 1 to 2 and MA3 domains 3 to 4 of *Chlamydomonas reinhardtii* and *Volvox carteri* with the intron positions indicated. The MA3 domains are indicated by the bold text and above the pertinent sequence. The presence of the intron between the two amino acid residues indicated in red with black type (see asterisk) in the same position in domains 1 and 3 of both species suggests domain duplication. Two additional introns with near identical positions are indicated in red with white type. The positions of non-conserved introns are indicated in blue with white type.

If the four MA3 domain PDCD4 homolog of *C. reinhardtii* and *V. carteri* arose from the duplication of a two MA3 domain progenitor, any intron present in the gene encoding the progenitor might also have been duplicated during the generation of the four MA3 domain form of the protein. The *C. reinhardtii* and *V. carteri* PDCD4 genes contain numerous introns some of which are shared between the two. To determine if any may have predated the duplication, the genomic sequence representing MA3 domains 1 to 2 was compared to MA3 domains 3 to 4 of the PDCD4 homologs for both algal species (Figure 4). One intron present in MA3 domain 1 of *C. reinhardtii* and *V. carteri* PDCD4 is present in precisely the corresponding location in the MA3 domain 3 of the same protein (see asterisk, Figure 4), suggesting that it was present in the ancestor of *C. reinhardtii* and *V. carteri* that contained a two MA3 domain PDCD4 progenitor gene that underwent duplication and resulted in a copy of the intron at the same location in MA3 domains 1 and 3. An intron just C-terminal to MA3 domains 1 and 3 and another in MA3 domains 2 and 4 are in near-identical positions but it is not possible to conclude that these predated the duplication event. Nevertheless, the identical position of an intron in MA3 domains 1 and 3 of *C. reinhardtii* and *V. carteri* PDCD4 supports the notion that the four MA3 domain form of their protein arose through duplication of a two MA3 domain PDCD4 progenitor during their evolution.

As land plants did not evolve from Chlamydomonadales but rather from charophytes (i.e., fresh water algae), the appearance of a four MA3 domain PDCD4 homolog in *C. reinhardtii* and *V. carteri* likely represents an independent event from the appearance of the four MA3 domain PDCD4 homolog in the land plant lineage. This is because charophytes and chlorophytes (which include *C. reinhardtii* and *V. carteri*) are thought to have derived from a common ancestor that likely contained a two MA3 domain PDCD4 form of the protein that was maintained in older algal lineages (e.g., those in Mamiellales) but underwent duplication during later chlorophyte evolution.

**Figure 5:**
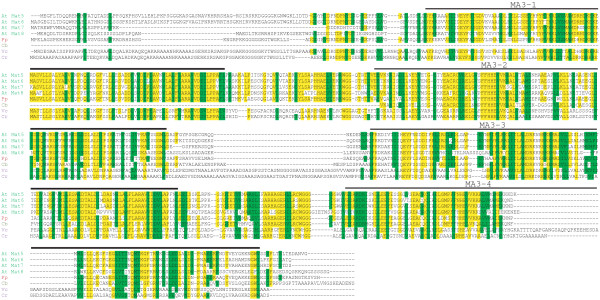
**Sequence alignment of four MA3 domain PDCD4 homologs in algae and land plants.** Alignment of PDCD4 amino acid sequence is shown with amino acid identity relative to *A. thaliana* MAT5 highlighted in yellow amino acid and similarity highlighted in green. Each MA3 domain present in all proteins is indicated above the pertinent sequence. Protein sequences used were: At MAT5, *Arabidopsis thaliana* GenBank: NP_567708; At MAT6, GenBank: NP_568968; At MAT7, GenBank: NP_190411; At MAT8, GenBank: NP_173687; Pp, *Physcomitrella patens* GenBank: XP_001777050; Cb, *Chaetosphaeridium globosum* GenBank: HO379087; Vc, *Volvox carteri* GenBank: XP_002956063; and Cr, *Chlamydomonas reinhardtii* GenBank: XP_001696827.

Thus, examination of charophyte PDCD4 homologs might more precisely determine when the four MA3 domain form of the protein appeared during the evolution of land plants. Although little genome sequence information is available for charophytes, a search of the EST database identified a partial PDCD4 cDNA from *Chaetosphaeridium globosum*, representing the Coleochaetophyceae. Although the 5′-end of the cDNA is truncated, the predicted protein contains MA3 domains 2–4 and, unlike PDCD4 present in *C. reinhardtii* and *V. carteri*, the *C. globosum* homolog shares similarity to the regions between the MA3 domains of land plant PDCD4 homologs, particularly between the second and third MA3 domains (Figure 5) which represents the fusion site during the putative duplication of the two MA3 domain form of PDCD4. This similarity between *C. globosum* PDCD4 and higher plants is in stark contrast to the lack of similarity of the region between the second and third MA3 domains of *C. reinhardtii* and *V. carteri* PDCD4 with higher plants, both in sequence and in length (Figure 5). Moreover, the fourth MA3 domain of the *C. reinhardtii* and *V. carteri* PDCD4 is interrupted by a sequence not present in *C. globosum* or higher plant PDCD4 homologs. Although this insertion likely occurred subsequent to the duplication event that gave rise to the four MA3 domain PDCD4 homolog in these chlorophytes, the PDCD4 of the Chlamydomonadales does not contain four uninterrupted MA3 domains as a consequence. These data suggest, therefore, that the appearance of a PDCD4 protein with four uninterrupted MA3 domains predated the colonization of land and occurred during charophyte evolution.

### The PDCD4 gene family underwent expansion during land plant evolution

The PDCD4 homolog in *C. reinhardtii* and *V. carteri* is encoded by a single gene as it appears to be in other algae and algal relatives (Table 1). The PDCD4 gene family had expanded to two members by the appearance of early land plants such as *P. patens* and *S. moellendorffii*. Two members are also present in several dicotyledonous species such as *Vitis vinifera*, *Populus trichocarpa*, *Ricinus communis*, *Medicago truncatula*, *Glycine max*, and *Theobroma cacao* whereas three members are present in other dicots such as *Solanum lycopersicum*, *Gossypium raimondii*, *Thellungiella halophila*, and *Brassica rapa* (Table 1). Similarly, two members are present in some monocotyledonous species such as *Hordeum vulgare* and *Zea mays* while other monocots have three members such as *Sorghum bicolor*, *Brachypodium distachyon*, and *Oryza sativa* (Table 1). In contrast to *T. halophila*, and *B. rapa*, the PDCD4 gene families of *A. thaliana*, *A. lyrata*, and *Capsella rubella* are even larger with each containing four members. The variable size of the gene family among angiosperm species suggests that the expansion from the two gene members present in *P. patens* and *S. moellendorffii* occurred in a species-specific manner.

**Table 1:** List of plant species and PDCD4 sequences used with the number of MA3 domains present in each protein

**Organism**	**Protein accession**	**Nucleotide accession**	**Number of MA3 domains**
*Arabidopsis thaliana*	NP_567708	At4g24800 (MAT5)	4
	NP_568968	At5g63190 (MAT6)	4
	NP_190411	At3g48390 (MAT7)	4
	NP_173687	At1g22730 (MAT8)	4
*Capsella rubella*	EOA15859		4
	EOA14564		4
	EOA23591		4
	EOA39121		4
*Thellungiella halophila*	Thhalv10024556m^a^		4
	Thhalv10003735m^a^		4
	Thhalv10006903m^a^		4
*Brassica rapa*	Bra013836^a^		4
	Bra038615^a^		4
	Bra024534^a^		4
*Gossypium raimondii*	Gorai.001G062000.1^a^		4
	Gorai.003G108800.1^a^		4
	Gorai.005G076400.1^a^		4
*Theobroma cacao*	Thecc1EG014544t1^a^		4
	Thecc1EG039086t1^a^		4
*Populus trichocarpa*	XP_002318100	XM_002318064	4
	XP_002307530	XM_002307494	4
*Ricinus communis*	XP_002511272	XM_002511226	4
	XP_002527108	XM_002527062	4
*Medicago truncatula*	XP_003608913	XM_003608865	4
	XP_003619027	XM_003618979	4
*Glycine max*	XP_003525619	XM_003525571	4
	XP_003548962	XM_003548914	4
*Vitis vinifera*	XP_002277813	XM_002277777	4
	XP_002264439	XM_002264403	4
*Solanum lycopersicum*	XP_004237843	LOC101258853	4
	XP_004236308	LOC101255979	4
	XP_004236309	LOC101256273	4
*Eschscholzia californica*	TUHA-2426^b^		4
	EVOD-8494^b^		4
*Aristolochia elegans*	PAWA5255^b^		4
	PAWA2930^b^		4
*Hordeum vulgare*	BAJ94459	AK363255	4
	BAK08048	AK376854	4
*Sorghum bicolor*	XP_002444968	Sb07g002090	4
	XP_002448072	Sb06g020520	4
	XP_002465627	Sb01g042530	4
*Brachypodium distachyon*	XP_003573315	XM_003573267	4
	XP_003579994	XM_003579946	4
	XP_003561877	XM_003561829	4
*Zea mays*	NP_001147914	NM_001154442	4
	NP_001159302	NM_001165830	4
*Oryza sativa*	NP_001060879	Os08g0120500	4
	NP_001053119	Os04g0482800	4
	AAN05329	Os03g0222100	4
*Illicium floridanum*	VZCI-11530^b^		4
*Amborella trichopoda*	URDJ-37850^b^		4
*Austrobaileya scandens*	FZJL-1758^b^		4
	FZJL-1759^b^		4
*Picea abies*	MA_2314g0010^c^		4
	MA_10693g0010^c^		4
*Cycas micholitz*	XZUY-1711^b^		4
*Sundacarpus amarus*	KLGF-13902^b^		4
*Selaginella moellendorffii*	XP_002967718	XM_002964270	4
	XP_002979104	XM_002979058	4
*Physcomitrella patens*	XP_001777050	XM_001776998	4
	XP_001754785	XM_001754733	4
*Chaetosphaeridium globosum*	HO379087		4^d^
*Volvox carteri*	XP_002956063	XM_002956017	4
*Chlamydomonas reinhardtii*	XP_001696827	XM_001696775	4
*Chlorella variabilis*	EFN55681	GL433844	4^a^
*Ostreococcus tauri*	XP_003079341	XM_003079293	2
*Ostreococcus lucimarimus*	XP_001417999	XM_001417962	2
*Micromonas pusilla*	XP_003056900	XM_003056854	2
*Micromonas* sp. RCC299	XP_002502542	XM_002502496	2
*Phaeodactylum tricornutum*	XP_002184928	XM_002184892	2
*Ectocarpus siliculosus*	CBJ33138	FN648632	2
*Thalassiosira pseudonan*	XP_002295291	XM_002295255	2
*Aureococcus anophagefferens*	EGB07675	GL833130	2
*Phytophthora sojae*	EGZ06926	JH159163	2
*Phytophthora infestans*	XP_002899252	XM_002899206	2
*Albugo laibachii*	CCA24083	FR824266	2

^**a**^Obtained from Phytozome v9.1 [38].

^**b**^Obtained from http://www.onekp.com.

^**c**^Obtained from the Spruce Genome Project [39].

^**d**^Partial sequence available suggests the likely presence of four MA3 domains.

Gene divergence often follows gene duplication. To determine how the plant PDCD4 gene family members may have diverged during the evolution of land plants, phylogenetic analysis of the gene family was performed. Because of the poor sequence conservation of the N-terminus, only the region containing the four MA3 domains was used for the analysis. Although the *C. globosum* sequence is not full-length, it was included to represent a progenitor sequence to land plants. Similarly, one of the *P. abies* PDCD4 homologs (MA_10693g0010) is not full-length but was included along with the second *P. abies* homolog (MA_2413g0010), which is full-length, to represent the two PDCD4 gene members of a more recent gymnosperm species.

**Figure 6:**
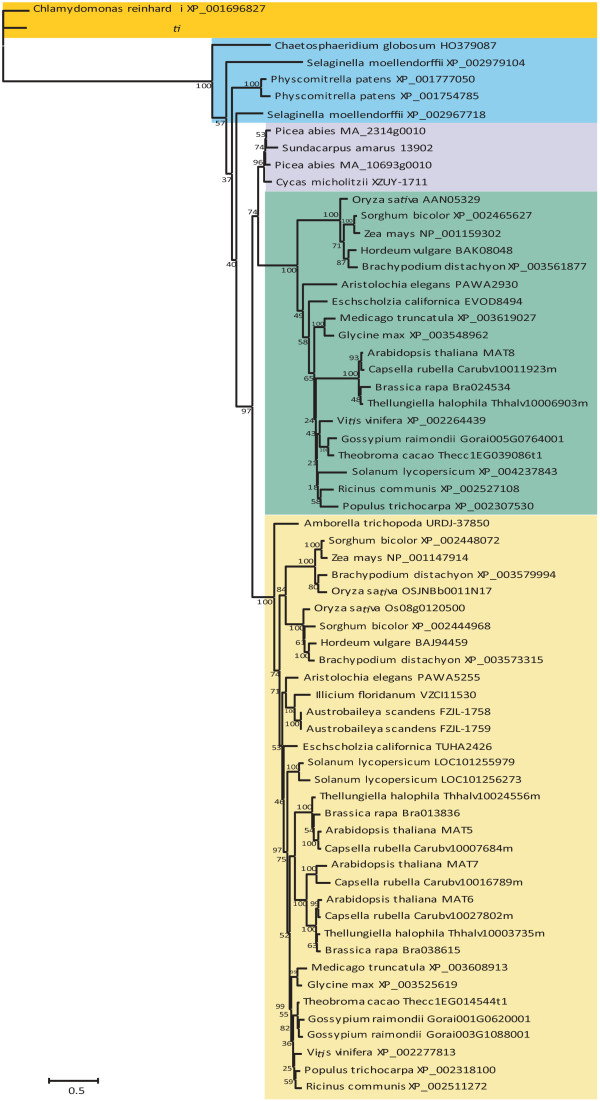
**Phylogeny of PDCD4 homologs in higher and lower plants.** Phylogenetic analysis of the aligned sequence comprising MA3 domains 1 to 4 for the four MA3 domain PDCD4 proteins of the plant species indicated. The phylogenetic tree was constructed using the maximum-likelihood method. Numbers on each branch denote percentages of bootstrap support. The MAT5/6/7 subgroup of monocots and dicots is shaded yellow, the MAT8 subgroup is shaded green, the PDCD4 homologs in gymnosperms is shaded purple, while those of early land plants and *Chaetosphaeridium globosum* are shaded blue and those in marine algae are shaded orange. Only partial sequences were available for *C. globosum* and *P.abies* MA_10693g0010.

The single PDCD4 gene family members of *C. reinhardtii* and *V. carteri* clustered together but not with the *C. globosum* sequence (Figure 6), which itself is more closely related to the PDCD4 homologs of land plants. This observation supports the notion that the four MA3 domain PDCD4 arose independently in chlorophytes and charophytes and that the PDCD4 homolog of land plants evolved from charophytes. The two PDCD4 homologs of *P. patens* clustered together but the two *S. moellendorffii* gene members exhibited some degree of divergence as they did not cluster together (Figure 6). In angiosperms, the PDCD4 gene family clusters into two distinct orthologous groups (Figure 6). One is represented by the *A. thaliana* MAT8 gene (the MAT8 subgroup) which is more closely related to MAT8-like gene members in other dicots such as *C. rubella*, *T. halophila*, *B. rapa*, *G. raimondii*, *T. cacao*, *G. max*, *M. truncatula*, *R. communis*, *P. trichocarpa*, *V. vinifera*, and *S. lycopersicum*, as well as in monocots such as *O. sativa*, *S. bicolor*, *Z. mays*, *H. vulgare*, *B. distachyon* than it is to the other three members of the *A. thaliana* PDCD4 gene family (i.e., MAT5, MAT6, and MAT7), suggesting that genes in the MAT8 cluster are true orthologs. Regardless of the size of the MAT gene family, only one MAT8 gene is observed in those species in which a MAT8 homolog is present. In many species examined, the PDCD4 gene family is composed of just two members so that there is a single member in each subgroup. In some species, however, there are more than two PDCD4 genes and those that are not similar to MAT8 clustered together. An example is the *A. thaliana* gene family which, in addition to the single MAT8 gene, contains three additional members (i.e., the MAT5/6/7 subgroup), that are more closely related to one another than to any MAT8 homolog. As four members are present in *C. rubella* (Figure 6) and *A. lyrata* (data not shown), which cluster similarly to the *A. thaliana* gene family members, the MAT5/6/7 subgroup likely underwent gene expansion to three members from the two members of the MAT5/6/7 subgroup present in *T. halophila*, and *B. rapa* (Figure 6). The MAT5 and MAT6 subclades contain orthologs from *A. lyrata* (data not shown), *C. rubella*, *T. halophila*, and *B. rapa* (Figure 6) whereas the MAT7 subclade contains orthologs only from *A. lyrata* and *C. rubella*, data suggesting that MAT7 is likely the newest member of the MAT5/6/7 subgroup that appeared after the separation of *Arabidopsis/Capsella* from *Brassica*. The two *G. raimondii* PDCD4 homologs in the MAT5/6/7 subgroup exhibit considerable similarity (Figure 6), suggesting a relatively recent gene duplication event that produced these paralogs. A similar expansion of the PDCD4 gene family occurred in some monocot species such as *O. sativa*, *S. bicolor*, and *B. distachyon*, each of which contains two genes within the MAT5/6/7 subgroup (Figure 6). In these species, however, the two gene members in the MAT5/6/7 subgroup cluster into two distinct clades, suggesting that gene duplication occurred prior to speciation.

These data suggest that the two genes produced from the duplication of the original PDCD4 gene during early higher plant evolution might have been the progenitor genes for MAT8 and the MAT5/6/7 subgroups and the latter subgroup underwent expansion in some species. However, the considerable similarity of the two PDCD4 homologs in *P. patens* suggests that gene duplication occurred relatively recently in this species. In contrast, the two homologs in *S. moellendorffii* have diverged suggesting an earlier gene duplication event. The two gene members of *P. patens* and *S. moellendorffii* are sufficiently different from the MAT8 and MAT5/6/7 subgroups that it is not possible to assign them to either subgroup. Moreover, the gymnosperm PDCD4 homologs cluster in a subclade that is separate from the MAT8 and MAT5/6/7 subgroups, including the two homologs of *P. abies*, suggesting that the gymnosperm PDCD4 homologs have diverged from angiosperms homologs. No MAT8 homolog was identified in basal angiosperm species as the PDCD4 homologs in the basal angiosperms *Amborella trichopoda*, *Austrobaileya scandens*, and *Illicium floridanum* are more similar to the MAT5/6/7 subgroup than the MAT8 subgroup (Figure 6). A MAT8-like homolog was identified in *Aristolochia elegans* and *Eschscholzia californica* in addition to MAT5/6/7-like homologs (Figure 6). These observations suggest that MAT8 subgroup is not present in basal angiosperms but appeared at or prior to the evolution of species within the Piperales such as *A. elegans*.

**Figure 7:**
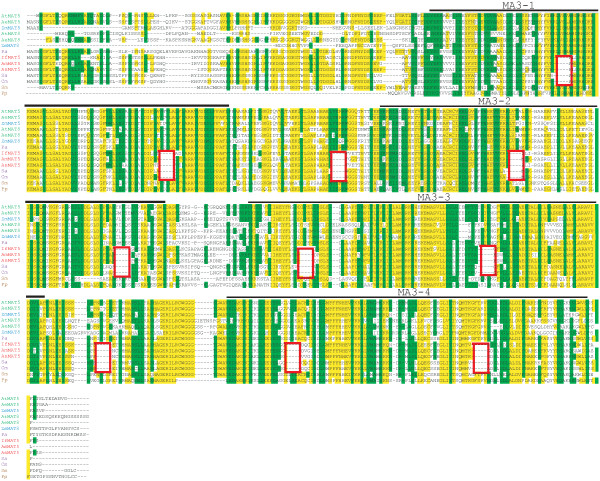
**Comparison of basal angiosperm and gymnosperm PDCD4 homologs with other land plants.** Alignment of PDCD4 amino acid sequence is shown with amino acid identity relative to *A. thaliana* MAT5 highlighted in yellow and similarity highlighted in green. Each MA3 domain present in all proteins is indicated above the pertinent sequence. The deletions present in *Cycas micholitz*, *Sundacarpus amarus*, *Illicium floridanum*, *Amborella trichopoda*, *Austrobaileya scandens* are outlined in red. Protein sequences used were: At MAT5, *Arabidopsis thaliana* GenBank: NP_567708; At MAT8, GenBank: NP_173687; Ae MAT5, *Aristolochia elegans* PAWA5255; Ae MAT8, *Aristolochia elegans* PAWA2930; Zm MAT5, *Zea mays* GenBank: NP_001147914; Zm MAT8, *Zea mays* GenBank: NP_001159302; Pa, *Picea abies* MA_2314g0010; If MAT5, *Illicium floridanum* VZCI-11530; Am MAT5, *Amborella trichopoda* URDJ-37850; As MAT5, *Austrobaileya scandens* FZJL-1758; Cm, *Cycas micholitz* XZUY-1711; Sa, *Sundacarpus amarus* 13902; Sm, *Selaginella moellendorffii* GenBank: XP_002979104; Pp, *Physcomitrella patens* GenBank: XP_001754785.

The PDCD4 homologs in *Cycas micholitz* and *Sundacarpus amarus* contain deletions in each MA3 domain relative to the early land plants and angiosperms (Figure 7), perhaps suggesting a unique feature of gymnosperm PDCD4 homologs. However, examination of the *P. abies* and *P. taeda* PDCD4 homologs revealed that they are similar to more recently evolved angiosperm species in that the deletions present in *C. micholitz* and *S. amarus* are not present in the MA3 domains of *P. abies* (Figure 7) and *P. taeda* (data not shown). Moreover, examination of the PDCD4 homologs of the basal angiosperms *A. trichopoda*, *A. scandens*, and *I. floridanum* revealed that they too contain deletions in each MA3 domain that are identical to those observed in *C. micholitz* and *S. amarus* (Figure 7). However, the PDCD4 homologs of *A. elegans* and more recently evolved angiosperm species do not share these deletions (Figure 7).

**Figure 8:**
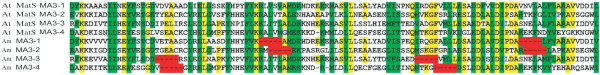
**Comparison of the MA3 domains of *A. thaliana *with those of the basal angiosperm *A. trichopoda.*** The individual MA3 domains of *A. thaliana* MAT5 (GenBank: NP_567708) and the basal angiosperm *A. trichopoda* (URDJ-37850) were aligned to show the relative positions of the deletions (highlighted in red) present in the MA3 domains of *A. trichopoda* PDCD4. Amino acid identity is highlighted in yellow, similarity is highlighted in green.

To examine if the deletions in each MA3 domain affect the same region, the individual MA3 domains from *A. thaliana* MAT5 and the basal angiosperm *A. trichopoda* were aligned. Nearly the same two regions deleted in the first MA3 domain of *A. trichopoda* were also missing in the second MA3 domain (Figure 8). Similarly, nearly the same two regions deleted in the third MA3 domain of *A. trichopoda* were also missing in the fourth MA3 domain, although these were not conserved with the deleted regions in the first two MA3 domains (Figure 8). As the early evolved gymnosperm species share precisely the same deletions as the basal angiosperm species, the observations of *A. trichopoda* apply to these gymnosperm species as well.

As these deletions are not present in the early land plants *P. patens* and *S. moellendorffii* but appear in the early lineage of both gymnosperms and angiosperms only to disappear again during subsequent evolution of each lineage, the most parsimonious explanation is that the deletions in the MA3 domains of early evolved gymnosperm and angiosperm species likely occurred independently in the evolution of these species as they branched from the ancestral gymnosperm and angiosperm lineages. It is also formally possible that the deletions appeared subsequent to early land plant evolution but prior to the appearance of the distinct gymnosperm and angiosperm lineages only to disappear again during the subsequent evolution of each lineage although this possibility would seem less likely. Despite the deletions present in the basal angiosperm PDCD4 homologs, they are more similar to the MAT5/6/7 subgroup than the MAT8 subgroup (Figure 6), supporting the conclusion that the MAT8 member of the PDCD4 gene family evolved following basal angiosperm evolution and is specific to more recently evolved angiosperm species.

## Discussion and conclusions

These results suggest that the domain organization of PDCD4 homologs in most lower plants, i.e., algae and algal relatives, is similar to those in animals in that it contains two MA3 domains but it underwent duplication to a four MA3 domain form in some recently evolved chlorophytes such as *C. reinhardtii* and *V. carteri* and, independently, in at least one charophyte such as *C. globosum*. That land plants did not evolve from chlorophytes, particularly from those species that contain a four MA3 domain PDCD4 homolog, supports the possibility of an independent duplication event during charophyte evolution. The sequence similarity between higher plant and *C. globosum* PDCD4 homologs at the fusion site between MA3 domain 2 and 3 and the significant sequence difference between higher plant and *C. reinhardtii* and *V. carteri* PDCD4 homologs in this same region further supports this conclusion. In addition to the duplication of PDCD4 from a protein containing two MA3 domains to a protein containing four MA3 domains, the PDCD4 gene family expanded from a single member in algae to two or more members upon colonization of land and the evolution of higher plants. The expansion of the gene family occurred early during higher plant evolution as evidenced by the two member gene family present in bryophytes and lycophytes with additional expansion of the gene family in some higher plant species that appears to have occurred in a species-specific manner.

The PDCD4 homologs of the angiosperm species examined have diverged into MAT8-like and MAT5/6/7-like subgroups. No MAT8 homolog was identified in gymnosperm or basal angiosperm species, but appeared at or prior to the evolution of species within the Piperales such as *A. elegans*, suggesting that the MAT8 homolog is an angiosperm-specific member that evolved after the appearance of the most basal angiosperm species. Although no further expansion of the gene family appears to have occurred within the MAT8 subgroup as only one gene encoding a MAT8-like homolog was observed in those species whose PDCD4 gene family included a MAT8-like member, additional expansion did occur in the MAT5/6/7 subgroup in some dicot and monocot species in a species-specific manner resulting in the paralogs observed in this subgroup. For example, orthologs of *A. thaliana* MAT7 are found in *A. lyrata* and *C. rubella* but not in *T. halophila* and *B. rapa*, suggesting it appeared following the separation of *Arabidopsis/Capsella* from *Brassica*. Other recent gene duplication events may have occurred in *P. patens* and *G. raimondii*. In contrast, the expansion of the MAT5/6/7 subgroup in some monocots such as *O. sativa*, *S. bicolor*, and *B. distachyon* predated their speciation as each contains two genes within the MAT5/6/7 subgroup which cluster into two distinct clades. All PDCD4 homologs identified in basal angiosperm species were MAT5/6/7-like, supporting the conclusion that this form of PDCD4 evolved prior to the appearance of MAT8-like homologs.

Interestingly, the PDCD4 homologs in early evolved gymnosperm and angiosperm species possess two deletions within each MA3 domain relative to early land plants. The observation that these deletions are present in both early evolved gymnosperm and angiosperm species supports the notion that they occurred prior to the appearance of the distinct gymnosperm and angiosperm lineages. However, the fact that these deletions are not observed in angiosperm species since (and including) the evolution of *A. elegans* and are not present in more recently evolved gymnosperm species such as *P. abies* and *P. taeda*, suggests that these deletions in the PDCD4 homologs of early evolved gymnosperm and angiosperm species likely occurred independently during the evolution of these species after they branched from the ancestral gymnosperm and angiosperm lineages. If they did occur independently in early gymnosperm and angiosperm species, it would suggest convergent evolution which may have been driven by the deletion of structural or functional elements within each MA3 domain that may have conferred an advantage to these species. The importance of the deleted regions within each MA3 domain is unknown but the elucidation of their structural or functional impact on the MA3 domains will provide insight into the adaption of PDCD4 proteins in early evolved gymnosperm and angiosperm species.

These observations collectively raise the question of what advantage does a four MA3 domain PDCD4 protein confer to recently evolved algal species and land plants and what advantage does expansion of the PDCD4 gene family confer to plants as they transitioned from an aquatic to a terrestrial environment? Loss of expression of just one of the four PDCD4 proteins in *A. thaliana* was sufficient to alter ethylene sensitivity and tolerance to salt [31]. The ability of MAT5 (ECIP1) to bind ethylene receptors and the EIN2 signaling component, suggests that plant PDCD4 may regulate ethylene signaling which in turn regulates responses to abiotic stresses such as salt [31]. Whether plant PDCD4 may interact also with eIF4A to inhibit protein synthesis as has been reported for animal PDCD4 remains to be determined. Moreover, its function in programmed cell death in plants has not been examined. An interesting correlation, however, is the presence in land plants and their closest algal relatives of a PDCD4 protein containing four uninterrupted MA3 domains with components involved in ethylene biosynthesis and signaling including homologs to the ETR2 and EIN4 ethylene receptors and the EIN2 downstream signaling component to which MAT5 binds [31,34,35]. This raises the intriguing possibility that the appearance of the four MA3 domain protein may represent the evolution of a protein involved in programmed cell death to one involved in abiotic stress-related hormone signaling similar to the evolution of ethylene receptors from two-component environmental sensor regulators [36]. The expansion of the PDCD4 gene family may have provided specificity of function if different PDCD4 isoforms are involved in different pathways or interact with different receptors within a single pathway such as in ethylene signaling. This would be particularly important following colonization of land which presents a more diverse array of stress conditions, such as desiccation, UV radiation, and temperature fluctuations, than would be present in many aquatic environments. *Arabidopsis* species contain five distinct ethylene receptors and MAT5 (ECIP1) interacts with just two of these, i.e., ETR2 and EIN4 (as well as EIN2). Whether other members of the PDCD4 gene family exhibit different specificities in their interactions with ethylene receptors, EIN2, or other proteins remains to be determined but the divergence of the MAT8 subgroup and the MAT5/6/7 subgroup might provide the basis for any functional specificity that exists within this family.

## Methods

### Sequence alignment and phylogenetic analysis

MA3-containing sequences obtained from *Arabidopsis thaliana* were used to query genome-wide analysis and comparative studies. MA3-containing sequences were obtained by BLAST searches of the NCBI [37] Phytozome v9.1 [38], and the Spruce Genome Project [39] protein, genome, and EST databases where appropriate. The Phytozome BLAST search implements NCBI Blast (v2.2.13). Reiterative searches of a particular species were performed using initial MA3-containing sequences from a species or from closely related species. Basal angiosperms and early evolved gymnosperm homologs were obtained from http://www.onekp.com. Predicted protein sequences from genomic and EST sequences were obtained using the ExPASy Translate tool [40]. Protein alignments were performed by Clustal Omega [41] with manual adjustments. Sequences queried included dicot and monocot plant genomes representing a diverse array of plants groups such as *Arabidopsis* relatives (*Capsella rubella*, *Thellungiella halophila*, and *Brassica rapa*), legumes (*Glycine max* and *Medicago truncatula*), the castor oil plant (*Ricinus communis*), cereals and grasses (*Zea mays*, *Sorghum bicolor*, *Oryza sativa*, *Hordeum vulgare*, and *Brachypodium distachyon*), fruits and vegetables (*Vitis vinifera* and *Solanum lycopersicum*), cotton (*Gossypium raimondii*), trees (*Populus trichocarpa* and *Theobroma cacao*), basal angiosperms (*Amborella trichopoda*, *Austrobaileya scandens*, and *Illicium floridanum*), gymnosperms (*Cycas micholitz*, *Sundacarpus amarus*, and *Picea abies*), bryophytes and lycophytes (*Physcomitrella patens* and *Selaginella moellendorffii*), green marine and fresh water algae (*Chlamydomonas reinhardtii*, *Volvox carteri, Chlorella variabilis*, *Micromonas* species, *Ostreococcus* species, and *Chaetosphaeridium globosum*), and stramenopiles or algal relatives (*Ectocarpus siliculosus*, *Aureococcus anophagefferens*, *Phaeodactylum tricornutum*, *Thalassiisira pseudoonana*, *Phytophthora* species, and *Albugo laibachii*). A list of protein sequences and cognate genes used for the comparative analysis is provided in Table 1. Maximum-likelihood phylogenetic trees were constructed using the PhyML software (v3.1) [42] with 1000 bootstrap replicates. The default LG amino acid replacement matrix [43] was used. Numbers included on each branch denote percentages of bootstrap support. Aligned sequences used for the phylogenetic analysis of Figure 2 are presented in Additional file 4; for the phylogenetic analysis of Additional file 3 in Additional file 5; and for the phylogenetic analysis of Figure 6 in Additional file 6.

## Abbreviations

ECIP1: EIN2 C-terminal interacting protein 1; eIF: Translation initiation factor; HEAT: Huntington, Elongation Factor 3, PR65/A, TOR; MAT: MA three; PABP: Poly(A) binding protein; PDCD4: Programmed cell death 4.

## Competing interest

The authors declared that they have no competing interest.

## Authors’ contributions

DG conceived of the study and directed its design and coordination. DG, RL, and SC contributed to the sequence alignment, phylogenetic analysis, and preparation of the manuscript. All authors read and approved the final manuscript.

## Supplementary Material

Additional file 1 **Sequence comparison of the MA3 domains in *A. thaliana *eIF4G and PDCD4 proteins.** Comparison of the amino acid sequence of the MA3 domains of eIF4G and PDCD4 proteins is shown with amino acid identity highlighted in yellow amino acid and similarity highlighted in green. Conserved residues for MAT5-8 proteins (MAT consensus) are indicated below the sequence alignment as are the conserved residues for eIF4G and eIFiso4G (4G/iso4G consensus). The eIFiso4G-like sequence is MAT4 (At4g30680). Those residues absolutely conserved among all MA3 domain proteins are indicated as bold residues in both consensus sequences. Click here for file

Additional file 2 **Domain organization of PDCD4 homologs in algae and algal relatives.** The domain organization of PDCD4 homologs is shown for green algae and algal relatives with the MA3 domains indicated in blue. PDCD4 proteins for green marine algae (*Chlamydomonas reinhardtii*, *Volvox carteri*, *Micromonas* species, and *Ostreococcus* species), fresh water alga (*Chaetosphaeridium globosum*), and stramenopiles or algal relatives (*Ectocarpus siliculosus*, *Aureococcus anophagefferens*, *Phaeodactylum tricornutum*, *Thalassiisira pseudoonana*, *Phytophthora* species, and *Albugo laibachii*) are shown. The additional sequence interrupting the fourth MA3 domain of the *C. reinhardtii* and *V. carteri* homologs is indicated by a gap. The first MA3 domain is missing from the partial *C. globosum* cDNA but is proposed. Click here for file

Additional file 3 **Sequence analysis of the MA3 domains of PDCD4 homologs in algae and algal relatives suggests domain duplication in at least some chlorophytes.** A phylogenetic tree was generated using the sequences comprising MA3 domains 1–2 and MA3 domains 3–4 of the four MA3 domain PDCD4 homologs of chlorophytes *Chlamydomonas reinhardtii* and *Volvox carteri,* and MA3 domains 1–2 and MA3 domain 3 from the partial cDNA of the *Chlorella variabilis* PDCD4 homolog, MA3 domains 1–2 of the two MA3 domain PDCD4 homologs of prasinophyte *Micromonas* species and *Ostreococcus* species, and those of the stramenopiles *Ectocarpus siliculosus*, *Aureococcus anophagefferens*, *Phaeodactylum tricornutum*, *Thalassiisira pseudoonana*, *Phytophthora* species, and *Albugo laibachii*. The phylogenetic tree was constructed using the maximum-likelihood method. Numbers on each branch denote percentages of bootstrap support. Chlorophytes are shaded dark green, species of the Mamiellales are shaded light green, and the stramenopiles are shaded tan. Click here for file

Additional file 4 **Aligned sequences used for the phylogenetic analysis of Figure**2**.** Click here for file

Additional file 5 **Aligned sequences used for the phylogenetic analysis of Additional file****3****.** Click here for file

Additional file 6 **Aligned sequences used for the phylogenetic analysis of Figure**6**.** Click here for file
