# Supplementary material for: The unique evolution of the programmed cell death 4 protein in plants
Source: BMC Evol Biol. 2013 Sep 16;13:199. doi: 10.1186/1471-2148-13-199 (PMC3850090; doi:10.1186/1471-2148-13-199)
Supplement: Additional file 1 — Sequence comparison of the MA3 domains in A. thaliana eIF4G and PDCD4 proteins. Comparison of the amino acid sequence of the MA3 domains of eIF4G and PDCD4 proteins is shown with amino acid identity highlighted in yellow amino acid and similarity highlighted in green. Conserved residues for MAT5-8 proteins (MAT consensus) are indicated below the sequence alignment as are the conserved residues for eIF4G and eIFiso4G (4G/iso4G consensus). The eIFiso4G-like sequence is MAT4 (At4g30680). Those residues absolutely conserved among all MA3 domain proteins are indicated as bold residues in both consensus sequences. [file 1471-2148-13-199-S1.pdf]

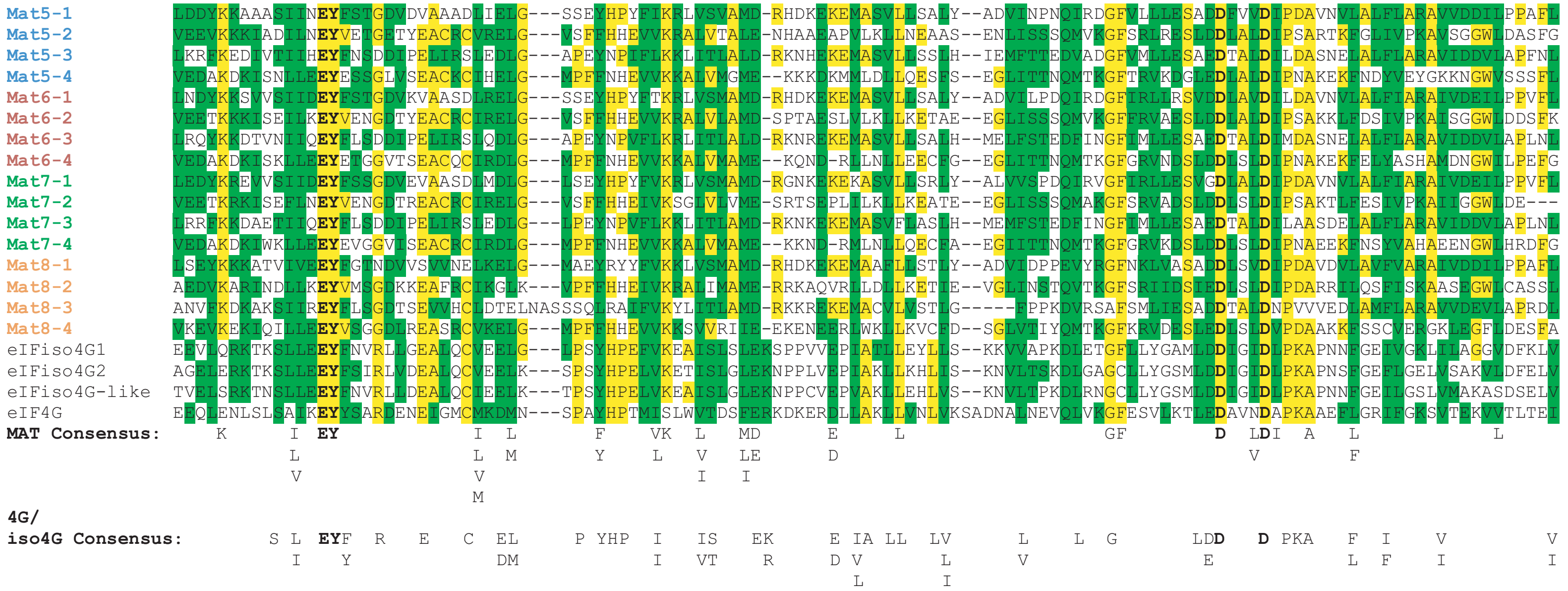

Sequence comparison of the MA3 domains in *A. thaliana* eIF4G and PDCD4 proteins. Comparison of the amino acid sequence of the MA3 domains of eIF4G and PDCD4 proteins is shown with amino acid identity highlighted in yellow amino acid and similarity highlighted in green. Conserved residues for MAT5-8 proteins (MAT consensus) are indicated below the sequence alignment as are the conserved residues for eIF4G and eIFiso4G (4G/iso4G consensus). The eIFiso4G-like sequence is MAT4 (At4g30680). Those residues absolutely conserved among all MA3 domain proteins are indicated as bold residues in both consensus sequences.
