# Supplementary material for: The unique evolution of the programmed cell death 4 protein in plants
Source: BMC Evol Biol. 2013 Sep 16;13:199. doi: 10.1186/1471-2148-13-199 (PMC3850090; doi:10.1186/1471-2148-13-199)
Supplement: Additional file 2 — Domain organization of PDCD4 homologs in algae and algal relatives. The domain organization of PDCD4 homologs is shown for green algae and algal relatives with the MA3 domains indicated in blue. PDCD4 proteins for green marine algae (Chlamydomonas reinhardtii, Volvox carteri, Micromonas species, and Ostreococcus species), fresh water alga (Chaetosphaeridium globosum), and stramenopiles or algal relatives (Ectocarpus siliculosus, Aureococcus anophagefferens, Phaeodactylum tricornutum, Thalassiisira pseudoonana, Phytophthora species, and Albugo laibachii) are shown. The additional sequence interrupting the fourth MA3 domain of the C. reinhardtii and V. carteri homologs is indicated by a gap. The first MA3 domain is missing from the partial C. globosum cDNA but is proposed. [file 1471-2148-13-199-S2.pdf]

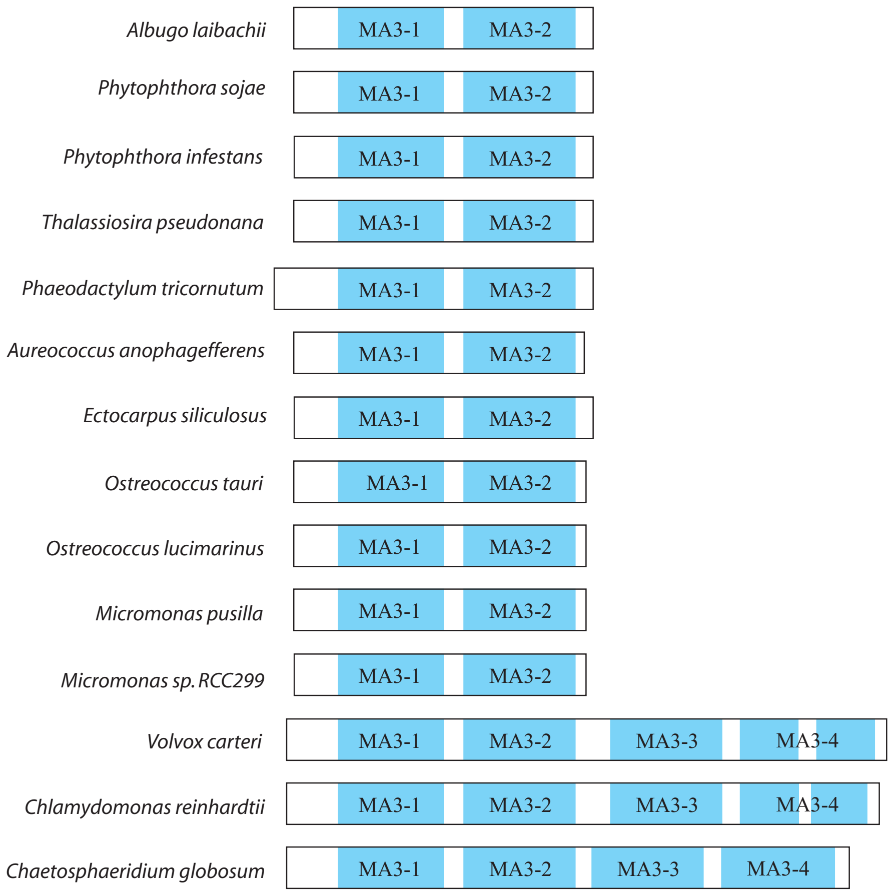

Domain organization of PDCD4 homologs in algae and algal relatives. The domain organization of PDCD4 homologs is shown for green algae and algal relatives with the MA3 domains indicated in blue. PDCD4 proteins for green marine algae (*Chlamydomonas reinhardtii*, *Volvox carteri*, *Micromonas* species, and *Ostreococcus* species), fresh water alga (*Chaetosphaeridium globosum*), and stramenopiles or algal relatives (*Ectocarpus siliculosus*, *Aureococcus anophagefferens*, *Phaeodactylum tricornutum*, *Thalassiisira pseudoonana*, *Phytophthora* species, and *Albugo laibachii*) are shown. The additional sequence interrupting the fourth MA3 domain of the *C. reinhardtii* and *V. carteri* homologs is indicated by a gap. The first MA3 domain is missing from the partial *C. globosum* cDNA but is proposed.

Additional file 2
