# Supplementary material for: The unique evolution of the programmed cell death 4 protein in plants
Source: BMC Evol Biol. 2013 Sep 16;13:199. doi: 10.1186/1471-2148-13-199 (PMC3850090; doi:10.1186/1471-2148-13-199)
Supplement: Additional file 3 — Sequence analysis of the MA3 domains of PDCD4 homologs in algae and algal relatives suggests domain duplication in at least some chlorophytes. A phylogenetic tree was generated using the sequences comprising MA3 domains 1–2 and MA3 domains 3–4 of the four MA3 domain PDCD4 homologs of chlorophytes Chlamydomonas reinhardtii and Volvox carteri, and MA3 domains 1–2 and MA3 domain 3 from the partial cDNA of the Chlorella variabilis PDCD4 homolog, MA3 domains 1–2 of the two MA3 domain PDCD4 homologs of prasinophyte Micromonas species and Ostreococcus species, and those of the stramenopiles Ectocarpus siliculosus, Aureococcus anophagefferens, Phaeodactylum tricornutum, Thalassiisira pseudoonana, Phytophthora species, and Albugo laibachii. The phylogenetic tree was constructed using the maximum-likelihood method. Numbers on each branch denote percentages of bootstrap support. Chlorophytes are shaded dark green, species of the Mamiellales are shaded light green, and the stramenopiles are shaded tan. [file 1471-2148-13-199-S3.pdf]

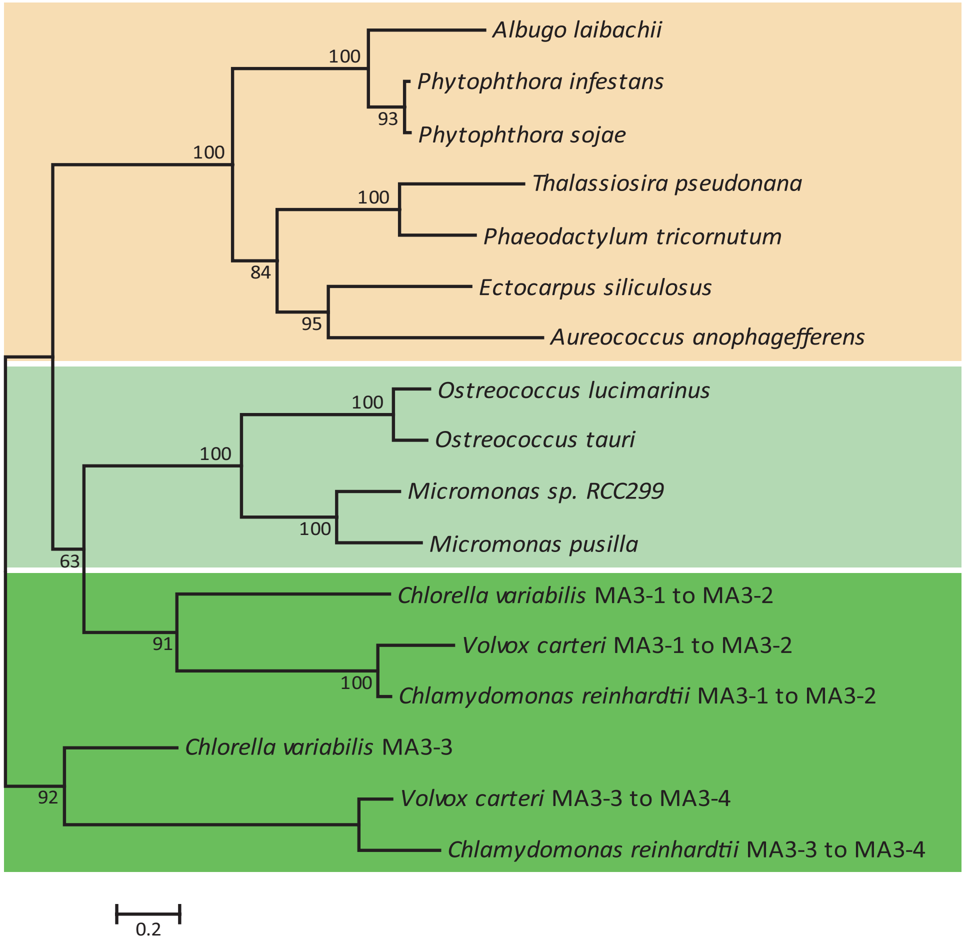

Sequence analysis of the MA3 domains of PDCD4 homologs in algae and algal relatives suggests domain duplication in at least some chlorophytes. A phylogenetic tree was generated using the sequences comprising MA3 domains 1-2 and MA3 domains 3-4 of the four MA3 domain PDCD4 homologs of chlorophytes *Chlamydomonas reinhardtii* and *Volvox carteri,* and MA3 domains 1-2 and MA3 domain 3 from the partial cDNA of the *Chlorella variabilis* PDCD4 homolog, MA3 domains 1-2 of the two MA3 domain PDCD4 homologs of prasinophyte *Micromonas* species and *Ostreococcus* species, and those of the stramenopiles *Ectocarpus siliculosus*, *Aureococcus anophagefferens*, *Phaeodactylum tricornutum*, *Thalassiisira pseudoonana*, *Phytophthora* species, and *Albugo laibachii*. The phylogenetic tree was constructed using the maximum-likelihood method. Numbers on each branch denote percentages of bootstrap support. Chlorophytes are shaded dark green, species of the Mamiellales are shaded light green, and the stramenopiles are shaded tan.
