# Supplementary material for: The unique evolution of the programmed cell death 4 protein in plants
Source: BMC Evol Biol. 2013 Sep 16;13:199. doi: 10.1186/1471-2148-13-199 (PMC3850090; doi:10.1186/1471-2148-13-199)
Supplement: Additional file 4 — Aligned sequences used for the phylogenetic analysis of Figure2. [file 1471-2148-13-199-S4.pdf]

Additional file 4. Aligned sequences used for the phylogenetic analysis of Figure 2.

>Mat5MA3-1

PLDDYKKAAASIINEYFSTGDVDVAAADLIELG---SSEYHPYFIKRLVSVAMDRHDKEK

EMASVLLSALYADVINPNQIRDGFVLLLESADDFVVDIPDAVNVLALFLARAVVDDILPP

AFL

>Mat5MA3-2

-VEEVKKKIADILNEYVETGETYEACRCVRELG---VSFFHHEVVKRALVTALENHAAEA

PVLKLLNEAASENLISSSQMVKGFSRLRESLDDLALDIPSARTKFGLIVPKAVSGGWLDA

S--

>Mat5MA3-3

-LKRFKEDIVTIIHEYFNSDDIPELIRSLEDLG---APEYNPIFLKKLITLALDRKNHEK

EMASVLLSSLHIEMFTTEDVADGFVMLLESAEDTALDILDASNELALFLARAVIDDVLAP

F--

>Mat5MA3-4

-VEDAKDKISNLLEEYESSGLVSEACKCIHELG---MPFFNHEVVKKALVMGME-KKKDK

MMLDLLQESFSEGLITTNQMTKGFTRVKDGLEDLALDIPNAKEKFNDYVEYGKKNGWVSS

SF-

>Mat6MA3-1

PLNDYKKSVVSIIDEYFSTGDVKVAASDLRELG---SSEYHPYFTKRLVSMAMDRHDKEK

EMASVLLSALYADVILPDQIRDGFIRLLRSVDDLAVDILDAVNVLALFIARAIVDEILPP

VFL

>Mat6MA3-2

-VEETKKKISEILKEYVENGDTYEACRCIRELG---VSFFHHEVVKRALVLAMDSPTAES

LVLKLLKETAEEGLISSSQMVKGFFRVAESLDDLALDIPSAKKLFDSIVPKAISGGWLDD

S--

>Mat6MA3-3

-LRQYKKDTVNIIQEYFLSDDIPELIRSLQDLG---APEYNPVFLKRLITLALDRKNREK

EMASVLLSALHMELFSTEDFINGFIMLLESAEDTALDIMDASNELALFLARAVIDDVLAP

L--

>Mat6MA3-4

--EDAKDKISKLLEEYETGGVTSEACQCIRDLG---MPFFNHEVVKKALVMAME-KQND-

RLLNLLEECFGEGLITTNQMTKGFGRVNDSLDDLSLDIPNAKEKFELYASHAMDNGWILP

EF-

>Mat7MA3-1

PLEDYKREVVSIIDEYFSSGDVEVAASDLMDLG---LSEYHPYFVKRLVSMAMDRGNKEK

EKASVLLSRLYALVVSPDQIRVGFIRLLESVGDLALDIPDAVNVLALFIARAIVDEILPP

VFL

>Mat7MA3-2

-VEETKRKISEFLNEYVENGDTREACRCIRELG---VSFFHHEIVKSGLVLVMESRTSEP

LILKLLKEATEEGLISSSQMAKGFSRVADSLDDLSLDIPSAKTLFESIVPKAIIGGWLDE

>Mat7MA3-3

-LRRFKKDAETIIQEYFLSDDIPELIRSLEDLG---LPEYNPVFLKKLITLAMDRKNKEK

EMASVFLASLHMEMFSTEDFINGFIMLLESAEDTALDILAASDELALFLARAVIDDVLAP

LN-

>Mat7MA3-4

-VEDAKDKIWKLLEEYEVGGVISEACRCIRDLG---MPFFNHEVVKKALVMAME-KKND-

RMLNLLQECFAEGIITTNQMTKGFGRVKDSLDDLSLDIPNAEEKFNSYVAHAEENGWLHR

DF-

>Mat8MA3-1

DLSEYKKKATVIVEEYFGTNDVVSVVNELKELG---MAEYRYYFVKKLVSMAMDRHDKEK

EMAAFLLSTLYADVIDPPEVYRGFNKLVASADDLSVDIPDAVDVLAVFVARAIVDDILPP

AFL

>Mat8MA3-2

-AEDVKARINDLLKEYVMSGDKKEAFRCIKGLK---VPFFHHEIVKRALIMAMERRKAQV

RLLDLLKETIEVGLINSTQVTKGFSRIIDSIEDLSLDIPDARRILQSFISKAASEGWLCA

S--

>Mat8MA3-3

-ANVFKDKAKSIIREYFLSGDTSEVVHCLDTELNASSSQLRAIFVKYLITLAMDRKKREK

EMACVLVSTLG---FPPKDVRSAFSMLIESADDTALDNPVVVEDLAMFLARAVVDEVLAP

R--

>Mat8MA3-4

-VKEVKEKIQILLEEYVSGGDLREASRCVKELG---MPFFHHEVVKKSVVRIIEEKENEE

RLWKLLKVCFDSGLVTIYQMTKGFKRVDESLEDLSLDVPDAAKKFSSCVERGKLEGFLDE

SF-
