# Supplementary material for: The unique evolution of the programmed cell death 4 protein in plants
Source: BMC Evol Biol. 2013 Sep 16;13:199. doi: 10.1186/1471-2148-13-199 (PMC3850090; doi:10.1186/1471-2148-13-199)
Supplement: Additional file 5 — Aligned sequences used for the phylogenetic analysis of Additional file3. [file 1471-2148-13-199-S5.pdf]

Additional file 5. Aligned sequences used for the phylogenetic analysis of Additional file 3.

>Micromonas_sp._RCC299
---YKNAVTAIIEEYFQTHDARETQRALDETNKPLYQHFFVKKLVTMSMDRGDREKEAAA
VLLSALYPHHVDPEQLQRGFERLLESVDDLAIDVPAAADDLAMFIAR-------------
--ATVDDILPPRFLHTNLEGLLPGLR-VGEKAAETIDLAHGHLHAHHGTERILRAWG-DS
DLTPLQQAKHAIQECLTEYV-SSGDVNEARRCLRSLHMNYFHHEFVKRALVLCIEAPEGH
-------------------------------ET-----APRLLGLLKVLG--KSGEVSAS
QMKI-GFDRMDVVVEDLVLDV-PKAK-TRLEGLRLMAKEEEIHPMRE

>Micromonas_pusilla
---YKASTASIVKEYFDSGDVAATAALLDDTEQPLYQHYFVKRLVTMSMDRGAKEKEAAA
VLLSALYPNHVSSLEIQRGFERLVESVDDLALDVPSAASDLAMFIAR-------------
--ATVDDILPPAFMHT-LEGLLPGLRGEGKHAFETLRIARGHLDGRHAHERVLRGFGVDS
SKSPIDAAKTAIQDLLTEYL-DSGDVAEARRCLRAINARYFHHEFVKRALVLCIEAVVGD
-------------------------------ET-----APRLLGLLKVLG--SSGEVSAS
QMAL-GFDRMAAVVEDLKLDV-PNAE-TRMEGLRLMAKEEGIHPMRD

>Ostreococcus_lucimarinus
---YKNKAETIINEYFNSADIDEAWISVEKLDAPVYEHFFVKRLVTLAMDRGHREKEAAA
TLLSALYPSALSGAQIQRGFLRLVEAADDLSIDVPDAAETLGMFIAR-------------
--AIIDDILPPSFPDN-VACL---VTCEGKQSQEALLLAHGHLFGPGHIDRVLRAWG-DF
DKSPLDAAKLQIKSMLEEYV-VTNDVSETRRCLHDLHMPFFHHEFVKQALNIALEAPRDC
-------------------------------HS-----VAVILGLFKVLG--DSAELSAS
QLQK-GFIRTNGAIEDLSLDI-PDAK-SKFDHIKQICVQHDV----V

>Ostreococcus_tauri
---YKRKAETIIDEYFNCSDIDEAWASVERLDAPVYEHFFVKRLVTLAMDRGNREKEAAA
TLLSALYPSALSGTQIQRGFVRLVESADDLAIDVPDTAEVLGMFIAR-------------
--AIIDDLLPPSFPDN-VAAM---DTCEGKTAQETLLLAHGHLTGPGHVDRVLRAWG-DF
DKSPLDAAKLQIKSMLDEYV-VTNDVSEIRHCLHDLHMAFFHHEFVKKALMLALEAPKDS
-------------------------------NI-----VANILGLLKVLG--DSAELSMS
QLQK-GYARVEGVIEDLSLDV-PDAK-SKLEHVKNICVQHDI----V

>Chlorella_variabilis_MA3-1_to_MA3-2
---FKKAVAMLLEEYYNSGDLNEAA----ELDRPEFGHYFVKRALATALDKHDREREMTS
VLLSTLYNEVIVPSQVRKGFMAAIDAMDDLKLDVPDVVDQLALFICRCAGRAGWCWCWGA
ACGVLDG--PPA-----------------SLGAELQAKCGLHLGAKHSGERLQRCWG-SG
AGFKFDETKQSIRSMLQEYA-SSGDKEEVARILRDLAVPFFHHELVKQALLLGMEAASQE
--AWLALLGKLS--ETGEVSAS
QMTKVGLAGARLCVPGT-----PGTPGAELNGTAHTPFHPSV----Q

>Volvox_carteri_MAT3-1_to_MAT3-2
---YKEQVRSIVEEYFVSGSVSDVAESLEELGASHLAHYFVKRLLTTALDHKDREREMAS
TLLSSLYAEVIAPDQLIKGFTSLFTSLPDLVLDVPEAPELLSRFVMR-------------
--AVVDDVLPPAIVSY-VDPE------SGPACRDLRQRCEAQLAARHNAEKVLRCWGGAG
TGTHFTDSKAAISSLLAEYL-VARDLGEASRRLRELGLPFFHHELVKQALVAALDNPSHV
---------------------------------------DPVVALLARLS--SSGEVSCS
QLAK-GLRRVADNLADAVLDN-PAAG-ERFAQLVAAARTAKV----F

>Chlamydomonas_reinhardtii_MAT3-1_to_MAT3-2
VAAYKEAVRALVEEYFSSGSVPDVVEGLEELGASHLAHYFVKRLITTALDRKDREREMAS
TLLSGLYAEVIAPEQVAKGFSSLFAALPDLVLDVPDAPELLCRFVTR-------------
--AVVDDVLPPAILSH-IDPE------ADPSCRDLRQRCETQLAARHSAEKVLRCWGGSG
AGTSHTDTKAAISSLLAEYLGASRDVAEAARRLRELGVPFFHHELVKQALLAAIESAANV

```
---------------------------------------DSVVALLGRLS--STGEVSAS
QLAK-GLRRVADNLADAVLDN-PQAG-ERFAALMGAAAAAKL----F

>Ectocarpus_siliculosus
---FKRHLKTIIEEYFLSEDISEVLRSVKELKSPAYHYEIVKRGINMSIDAKDHERELVS
KLLSDAYPDILSSREVCKGFERLFEMIDDIQLDAPNARTLVASFLAR-------------
--AVADEIIPPSVLRN-AAFL--------SLGGEIVKGARRLLSRDHVLSRLEHVWG-PG
DGRPVEELKVAIDQLLVEYL-LSRQQDEAAACVKELDCSLFHHEIVKRAVKAALDKTDDD
-------------------------------RT-------AMSSLLAYLN--KNEVISDE
QSKK-GFDRLHEILPDLVLDT-PAAP-SLLTKFTQQAISDGC----L

>Thalassiosira_pseudonana
---FKIRVSDAVREYFDSSDADEVVRCIDELKCREYHPEVVKRAISLGLDEGPRERELVS
RLLACLHPNPLRDEEMEGGFEVLLDSIEDLVIDIPDAKAMVGSFLAR-------------
--AVVDEVLAPAFLSN-RNNT--------HPGDCVVEKAVSLLSREHCTARLEKVWG-PG
DGRPVSELKDIMDQLLKEYL-LSRELDEAASCVRELKASHFNHELVKRGVKIAMEEDGRD
-------------------------------HASESSALDAMAALFKFLV--KNSIVSEY
QVAK-GVSRLRKIMPDLKLDV-PAAE-RMLDEFEGMA-KEGG----F

>Phaeodactylum_tricornutum
---FKVQVAEALKEYFDSCDADEVIRTLEELGCQEFHHEIVKKAISLAMDNSSRERELTS
RLLTCLHPTPLSMEHMEAGFNLLLDSVDDLSTDVPEAETMVASFLAR-------------
--AVVDEVLPPAYLSE-QNNV--------RVGDMVIAKAVALLSREHCTARLERVWG-PG
DGRPVEELKIEMDQLLQEYL-HSRELDEAARCVKELHAPHFHHELVKRGAFAAMELDGKK
-------------------------------EEQDHANLDAMAALLAFLV--KNAIVSEY
QVKK-GLSRLKDVLPDMQLDV-PLAP-ALMEAFAGFCAEQGC----L

>Albugo_laibachii
---IKKVIVEILEEFFVSGDYDESREQIIEKVPDEFKYDLVKRAITIAMDKHDKEREMAS
RFLSELYLKGLTPSQIQGGFRRVLLLAEDLEIDIPSAKGMLAIFCAR-------------
--AVVDEIVPPNFLED-PFLL--------RYSSDIAAEAIKKLSIHHGTARMEKGWG-PG
DGRPVEELKIAIDQLTKEYI-LSRDLDEATRCVRELNEPYFHHELVKRGIANALEESGED
-------------------------------------NLLAMASLFEYLV--TQDIVSKS
QLLK-GFEKFQQILDEIVLDI-PAAR-LQFETITKRAINDGI----L

>Phytophthora_infestans
---IKKRIIEILEEYFTSGDADEVLSSLNDLDEPEFNYEVVKRAITMAMDKNDKERELAS
RLLSTLYLDGLTAGQVLMGFRRVLLLAGDLQIDIPTAKNMLAIFCAR-------------
--AVVDEILPPSFLED-PFIT--------RYAPEIAGEAIKKLSINHATARMEKAWG-PG
DGRPVEELKVAIDQLTKEYL-LSRDLEEAARCVRELNVPHFHHEVVKRGITNSLEEGGEA
-------------------------------------NSAAMASLLAYLV--SNEVVSTG
QLVK-GFERFKFVLDDVALDI-PNAA-ALFKDIVARGISDGI----L

>Phytophthora_sojae
---IKKRIIEILEEYFTSGDADEVLSSLNDLDEPEFNYEVVKRAITMAMDKNDKERELAS
RLLSALYLDGLTAGQVLMGFRRVLLLAGDLQIDIPTAKNMLAIFCAR-------------
--AVVDEILPPSFLED-PFIT--------RYAPEIAAEAIKKLSINHATARMEKAWG-PG
DGRPVEELKVAIDQLTKEYL-LSRDLEEAARCVRELNVPHFHHEVVKRGITNSLEEGGEA
-------------------------------------NSAAMASLLAYLV--SHEVVSTG
QLIK-GFERFKLVLDDVALDI-PNAA-ALFQDTVARGVSDGI----L

>Aureococcus_anophagefferens
---FKRRVVEALDELFASGDVDECVTSLVELSCPEFGFEVVKRGVSKAVDRRARECELVS
RLLSAACPALLQPRDVAKGFERLFEAMDDLVLDAPRAPLVVGDFLVR-------------
--CVVDEALPPAYLGD-RVFV--------ALGGDIVARARRLLSREHALSKFERIWG-PG
DGRESSELKKVVDMLLHEYL-ATKELPEAKRCVRELSAPRFGHEVVKRAVTLALPRSADD
-------------------------------RT-------AISALLKALVVDPDQILSTT
```

```
QAKL-GFGRLAEALPDLTCDV-PNAK-ALLDEF-------------L

>Volvox_carteri_MAT3-3_to_MAT3-4
---FKAASLAALREYFDSQDAEEVAARLVALEEPGLHPLFVKAAVSLALDRKDRERELVS
KLLVALVPEVISPEALAGGFTRLLAAADDLVLDVPDAVHLLSLFLGR-------------
--VVVDELLPPAFLTQ-VLPS----LDADGLGVAVVRSAGIMLAARHGFERLVNCW--HG
GALELGAVRQAIRAAIEEYG-TSGDVAEVARCLRELGASSFSHEAVVAAVELAFSRYHGK
ATTTTQAPGANGSAQPQPKEEHESDAGAAPSDGSLEAAAGPVVELLTALA--GQGVLSAT
QLTT-GIERVRAALSEEVMDYGPSSQ-QVLNWITERGLREGW----L

>Chlamydomonas_reinhardtii_MAT3-3_to_MAT3-4
---FKAVTLAAVREYFDSQDSAEVAARLKGLDEPGLHPLFVKAAVSLSLDRKDRERELVS
KLLVALTPSVIAHSALAAGFTRLLAAADDLVLDVPDAVHLLSLFLGR-------------
--AVVDELLPPAFLTQ-VLSS----LDAEGLGVAVVRNAGIMLGARHGIERLVNCW--HG
GALELGAVRQAIRDAIAEYG-TSNDVAEVARCLRDLDAAAYNHEAVVAAAELACNRYHGK
-TGGAAAAAANG---------HDS------GDAELEAAVAPVVLLLGALS--AQGVVSST
QMAA-GMSRIRSAVEQEVMDYGPAAR-KVLDQLVAAGKRDGW----M

>Chlorella_variabilis_MA3-3
--AFKTAALDIVREYFDSGDAGEVAHRLRELDEPGFHNIFVKHAIQLAMDRKDRERELVS
ALLPTLVPATISADQ---------------------------------------------
------------------------------------------------------------
----------------------------AERCLQNLNVPHFHHDFVARSLLAAFGSEGEA
---------------------------------------GKVLGLLQQLA--DSGEVSQT
QMAK-GFARVEARLADTALDL-ARAP-ELYQQYKQQALEQGWLPA-A
```
