# Supplementary material for: The unique evolution of the programmed cell death 4 protein in plants
Source: BMC Evol Biol. 2013 Sep 16;13:199. doi: 10.1186/1471-2148-13-199 (PMC3850090; doi:10.1186/1471-2148-13-199)
Supplement: Additional file 6 — Aligned sequences used for the phylogenetic analysis of Figure6. [file 1471-2148-13-199-S6.pdf]

Additional file 6. Aligned sequences used for the phylogenetic analysis of Figure 6.

>Volvox_carteri_XP_002956063

AYKEQVRSIVEEYFVSGSVSDVAESLEELGASHLAHYFVKRLLTTALDHKDREREMASTL

LSSLYAEVIAPDQLIKGFTSLFTSLPDLVLDVPEAPELLSRFVMRAVVDDVLPPAIVSYV

D---PESGPACRDLRQRCEAQLAARHNAEKVLRCWGGAGTGTHFTDSKAAISSLLAEYLV

-ARDLGEASRRLRELGLPFFHHELVKQALVAALDNP-SHVDPVVALLARLSSSGEVSCSQ

LAKGLRRVADNLADAVLDNPAAGERFAQLVAAARTAKVFDDLEPEDMGTNAALAVFGTPA

AADGASGPSSTCASNGGGGGAVASSSGGASAGGVVVAMPPGVAAFKAASLAALREYFDSQ

DAEEVAARLVAL--E-EPGLHPLFVKAAVSLALDRKDRERELVSKLLVALVPEVISPEAL

AGGFTRLLAAADDLVLDVPDAVHLLSLFLGRVVVDELLPPAFLTQVLPS----LDADGL-

GVAVVRSAGIMLAARHGFERLVNCWHGGALEL--------GAVRQAIRAAIEEYGTSGDV

AEVARCLRELGASSFSHEAVVAAVELAFSRYHGKATTTTQAPGANGSAQPQPKEEHESDA

GAAPSDGSLEAAAGPVVELLTALAGQGVLSATQLTTGIERVRAALSEEVMDYGPSSQQVL

NWITERGLREGWL

>Chlamydomonas_reinhardtii_XP_001696827

AYKEAVRALVEEYFSSGSVPDVVEGLEELGASHLAHYFVKRLITTALDRKDREREMASTL

LSGLYAEVIAPEQVAKGFSSLFAALPDLVLDVPDAPELLCRFVTRAVVDDVLPPAILSHI

D---PEADPSCRDLRQRCETQLAARHSAEKVLRCWGGSGAGTSHTDTKAAISSLLAEYLG

ASRDVAEAARRLRELGVPFFHHELVKQALLAAIESA-ANVDSVVALLGRLSSTGEVSASQ

LAKGLRRVADNLADAVLDNPQAGERFAALMGAAAAAKLFNDLEAEDVGDNAAAALAAAGV

AIGSAAASAGGAASTGASTPNASNGGAAPSGRGVSVAMPPGVSAFKAVTLAAVREYFDSQ

DSAEVAARLKGL--D-EPGLHPLFVKAAVSLSLDRKDRERELVSKLLVALTPSVIAHSAL

AAGFTRLLAAADDLVLDVPDAVHLLSLFLGRAVVDELLPPAFLTQVLSS----LDAEGL-

GVAVVRNAGIMLGARHGIERLVNCWHGGALEL--------GAVRQAIRDAIAEYGTSNDV

AEVARCLRDLDAAAYNHEAVVAAAELACNRYHGKTGGAAAAA----------------AN

GHDSGDAELEAAVAPVVLLLGALSAQGVVSSTQMAAGMSRIRSAVEQEVMDYGPAARKVL

DQLVAAGKRDGWM

>Sorghum_bicolor_XP_002465627

QFKRKATTIVEEYFSTDDVAATANELRELRVPCYHYYFVKKLVSVAMDRHDREKEMAAVL

LSSLYGDVVDRPQLCKGFCKLTESCDDLSVDTPDAVDILAVFVARAVVDDMLPPAFLAKQ

SACLPDGCKGAEVLRRAEKSYLSVPHHGEIVLQRWGGSKRI-TVEEAKAKISDILEEYLA

-AGDRCEALRCIRDLKIPFFHHDVVKRALVLAVERGGASEAHILDLLKSASEEGVINESQ

IAKGFDRLIDSLDDLTLDVPNARCLVQSVIHKASSEGWLCVSCLKPLPPEP---------

-------------------------------KKSSEVDDAAVRQFKAKAVLIIKEYFLTG

DIIEVLSWLEAENFSCCPSFNAIFVQKLVNAAMDRKSREKEMASVLLSSLC---MPPEDV

VAGFHLLIEAAEDAALDNPAIVEDLTMFFARSVVDEVIAPSDLEAMEEDASRVKADG-ST

GMLALRNARALLGAKLSAERILRCWGGG--GSGKAGWEL-EEVKDKIGKLLQEYDCGGDI

REACRCIKDLGMPFFHHEVVKKALVAIIEKRGK---------------------------

-----------DER-LWGLLSECYGRGLITPNQMTKGFDRVADCVDDLALDV-PDAAKQL

GCCIDRAKKEGWL

>Zea_mays_NP_001159302

QFKRKATTIVEEYFSTDDVAATANELRELRVPCYHFYFVKKLVSVAMDRHDREKEKAAVL

LSSLYGDVVDRPQLCKAFCKLTESCDDLSVDTPDAVDILAVFVARAVVDDMLPPAFLATQ

SARLPHGCKGAKVLRRAEKSYLSVPHHGEIVLQRWGGSKRI-TVEEAKAKISDILEEYLA

-GGDRSEALRCVRDLKIPFFHHDVVKRALVLAVERGRAAEGLILDLLKSASEEGVINESQ

ITKGFDRLIDSLDDLALDVPNARCLLKSVIHKASSEGWLSESCLKPLPPEP---------

-------------------------------KKGSEVDDATVRQFKEKAVLIIKEYFLTG

DIIEVMSWLEAENYSCCPSFNAIFVQKLVNAAMDRKSREKEMASVLLSSLC---MPPEDV

VAGFHLLIDAAEDAALDNPAIVEDLTMFFARSVVDEVIAPSDLEALEEDAGRVKADG-SA

GMLALRNAHALLGAKLSAERILRCWGGG--GSGKAGWEL-DEVKDKIGKLLQEYDCGGDI

REACRCIKDLAMPFFHHEVVKKALVAIIEKRGR---------------------------

-----------DER-LWGLLSECYGRGLITPNQMTKGFDRMADCVDDLALDV-PDAAKQL

GCCIERAKKDGWL

>Oryza_sativa_AAN05329

QFKRKATTILEEYFSTDDVAATANELRELRVPCYHYYFVKKLVSVAMDRHDREKEMAAVL

LSSLYGDVIDRPQVYKGFGKLAESCDDLSVDTPDAVDILAVFVARAIIDDILPPAFLAKQ

LTCLPEGCKGAEVLHRAEKSYLSVPHHGEIILQRWGGSKSI-TVEEAKAKIADILEEYLA

-AGDIGEACRCIRGLKISFFHHDIVKRALTLAMERGGGAEGHILDLLKSASDEGIINESQ

ITKGFNRLIDSVDDLTLDVPNARRLLKSMILKASSEGWLCASSLKPLGPEP---------

-------------------------------KKAVVEDDAAVRQFKAKTLSIIKEYFLTG

DIIEVMSSLEAENYACSSSYNAIFVKKLITSAMDRKSREKEMASVLLSSLG---MPPEDV

VSGFHLLIESAEDAALDNPAIVEDLTMFFARSVVDEVIAPSNLEKMEEEAGRGKPGGSST

GLLALRNARALLGAKLSAERILRCWGGG--ATGKAGWEL-DDVKDKIGRLLQEYDCGGDI

REACQCIKELGMPFFHHEVVKKALVAIMEKRGK---------------------------

-----------DER-LWGLLAECYGRGLITPNQMTKGFERVAGCVDDLALDV-PDAGKQL

CCYVERAKKGGWL

>Hordeum_vulgare_BAK08048

QFKRKATTIVEEYFSTDDVAATATELRELRVPCYHYYFVKKVVSVAMDRHDREKEMAAVL

LSSLYGDVIDRPQVYKGFCKLAESCDDLSVDTPDAVDILAVFVARAIVDDILPPAFLAKQ

LPCLPDGCKGAEVIRRAEKSYLSVPHHGEIILQRWGGIKSI-TVEEAKARIADILEEYLA

-AGDTAEAFRCIRELNIPFFHHDVVKRALVLAIERGGAAEGHILDLLKSASDQGVINESQ

IIKGFNRMIDSVDDLTLDVPNARCLLKSIILKASSEGWLCASSLKPLGSEP---------

-------------------------------KKV-VEDDPAVKRFKAKAVASIHEYFLTG

DIIESVSRLEAENSSCSCFFNAIFVKKLISFAMDRKNREKEMASVLLSSIC---MPPEHV

VAGFHLLVDCAEDAALDNPAIVEDLTMFFARSVVDEVIAPSDLEAVEEEAGRRKAAG-SP

GMLAVRNAHAMLGAKLSAERILRCWGGG--GTGKAGWEL-NEVKDKIGKLLQEYDSGGGV

REACRCIKELGMPFFHHEVVKKALVAIIEKRGK---------------------------

-----------DER-LWGLLSECYGRGLITPNQMTKGFQRVADCVDDLVLDV-PDAGEQL

GRYVERAKKGGWL

>Brachypodium_distachyon_XP_003561877

QFKKKAATIVEEYFSTDDVGATANELRELRVPCYHYYFVKKLVSVAMDRHDREKEMAAVL

LSSLYGDVIDRPQVYKGFSKLTESCDDLSVDIPDAVDILAVFVARAVVDDILPPAFLAKQ

LPCLPDGSKGAEVIHRADKSYLSVPHHGEIILQRWGGIKSI-TVEEAKAKIADILEEYLA

-AGDTAEAFRCIRDLKVPFFHHDVVKRALVLAVERGGAAEGRILNLLKAASDEGVINESQ

MIKGFNRLTDSVDDLTLDVPNARCLLKSIILKASSEGWLCASSLKPLGPEP---------

-------------------------------KKKAAVDDTAVRNFKAKALSIIQEYFLTG

DIIESVSSLQAQNKSCASSFNAIFVKKLVSAAMDRKNREKEMASVLLSALS---MPPDDV

VAGFHLLIDSAEDAALDNLAIVEDLAMFFARSVVDEVIAPSDLEALEEEAGRRKAAS-SP

GMLALRNAHALLGAKLSAERILRCWGGG--GGGKAGWEL-DEVKDKIGKLLQEYDCGGGV
REACRCIKELGMPFFHHEVVKKVLVAIIEKRGM---------------------------
-----------DER-LWGLLGECYGRGLITPNQMTKGFQRVADCIDDLALDV-PDAGEQL
GRCVERAKEGGWL

>Brassica_rapa_Bra024534

EYKKKATIIVEEYFSTNDVVSVANELKELGMAEYRYYFVKKLVSMAMDRHDKEKEMAAFL
LSTLYADVIDPPEVYRGFNKLVTSADDLSVDIPDAVDVLAVFVARAIVDDILPPAFLKKQ
MNLLPDDSKGVEVLRKAEKSYLAAPLHAEVVEKRWGGADNW-TAEDVKGRINELLKEYVM
-SGDKKEAFRCIKGLKVPFFHHEIVKRALIMAMERRK-AQEKLLELLKEATEVGLINSTQ
VTKGFSRIIDLIEDLSLDIPEARSVLQCFISKAASEGWLCASSLKTLSTDS---------
-------------------------------GEKLLE-NSKANVFKDKVKSIVREYFLSG
DALEVVHCLETELGACSSQLRAIFVKYLITLAMDRKKREKEMACVLLSSLD---FPAKDV
RKAFSMLIESADDTALDNPVVVEDLAMFLARAVVDEVLAPRDLDELLS------QPGSSV
GEKVIQTAKTLLKARLSGERILRCWGGGGVETNSPGCTA-SEVKEKIQVLLEEYVSGGDL
GEACRCVKELGMPFFHHEVVKKSVVRIIEEQEK---------------------------
-----------KER-VWKLLKVCFESGLVTIYQMTKGFKRVGESVEDLCLDV-PDAVEKF
KSCVVRAKVEGFL

>Thellungiella_halophila_Thhalv10006903m

EYKKKATIIVEEYFSTNDVVSVANELKELGMAEYRYYFVKKLVSMAMDRHDKEKEMAAFL
LSTLYADVIDPPEVYRGFNKLVASADDLSVDIPDAVDVLAVFVARAIVDDILPPAFLKKQ
MNLLPDDSKGVEVLKKAEKSYLATPLHAEVVEKRWGGTDNW-TAEDVKGRINDLLKEYVM
-SGDKKEAFRCIKGLKVPFFHHEIVKRALIMAMERQK-AQVRLLDLLKEAIEVGLINSTQ
VTKGFSRIIDSIEDLSLDIPEARCILQSFISKAASEGWLCASSLKSLSADP---------
-------------------------------GEKSLE-NSSANEFKDKAKSIVREYFLSG
DTSEVVHCLETELSASSGQLRAIFVKYLITLAMDRKKREKEMACVLVSSLG---FPPKDV
RKAFSMLIESADDTALDNPVVVEDLAMFLARAVVDEVLAPRDLEELLNQ---TPEPGSTA
GEKVIQMAKTLLKARLSGERILRCWGGGGIETNSPGATV-TEVKEKIQVILEEYVSGGDL
KEACRCVKELGMPFFHHEVVKKSVVRIIEEKEK---------------------------
-----------KER-LWKLLKVCFESGLVTIYQMTKGFKRVSESLEDLSLDV-PDAAEKF
SSCVESAKLEGFL

>Arabidopsis_thaliana_MAT8

EYKKKATVIVEEYFGTNDVVSVVNELKELGMAEYRYYFVKKLVSMAMDRHDKEKEMAAFL

LSTLYADVIDPPEVYRGFNKLVASADDLSVDIPDAVDVLAVFVARAIVDDILPPAFLKKQ

MKLLPDNSKGVEVLRKAEKSYLATPLHAEVVEKRWGGTDNW-TAEDVKARINDLLKEYVM

-SGDKKEAFRCIKGLKVPFFHHEIVKRALIMAMERRK-AQVRLLDLLKETIEVGLINSTQ

VTKGFSRIIDSIEDLSLDIPDARRILQSFISKAASEGWLCASSLKSLSADA---------

-------------------------------GEKLLE-NSSANVFKDKAKSIIREYFLSG

DTSEVVHCLDTELNASSSQLRAIFVKYLITLAMDRKKREKEMACVLVSTLG---FPPKDV

RSAFSMLIESADDTALDNPVVVEDLAMFLARAVVDEVLAPRDLEEVLNQ---TPEAGSSV

GEKVIQMAKTLLKARLSGERILRCWGGGGIETNSPGSTV-KEVKEKIQILLEEYVSGGDL

REASRCVKELGMPFFHHEVVKKSVVRIIEEKEN---------------------------

-----------EER-LWKLLKVCFDSGLVTIYQMTKGFKRVDESLEDLSLDV-PDAAKKF

SSCVERGKLEGFL

>Capsella_rubella_Carubv10011923m

EYKKKATIIVEEYFGTNDVVSVANELKELGMAEYRYYFVKKLVSMAMDRHDKEKEMAAFL

LSTLYARVIDPPEVYRGFNKLVASADDFSVDIPDAVDVLAVFVARAIVDDILPPAFLKKQ

LKLLPDNSKGVEVLRKAEKSYLATPLHAEVVEKRWGGTDNW-TAEDVKARINDLLKEYVM

-SGDKTEAFRCIKGLKVPFFHHEIVKRALIMAMERRK-AELRLLDLLKETIEVGLINSTQ

VTKGFSRIIDSIEDLSLDIPDARRILQSFISKAASEGWLCASSLKSLSADS---------

-------------------------------GEKLLE-NTSANVFKDKAKSIILEYFLSG

DTSEVEHCLETELNASSSQLRAIFVKYLITLAMDRKKREKEMACVLVSTLG---FPPKDV

RKAFSMLIESADDTALDNPVVVEDLAMFLARAVVDEVLAPRDLEEVLNQ---TPEAGSSV

GEKVIQMAKTLLKARLSGERILRCWGGGGTETNSPGSTV-QEVKEKIQILLEEYVSGGDL

REACRCVKELGMPFFHHEVVKKSVVRIIEEKEK---------------------------

-----------EER-LWKLLKVCFDSGLVTIYQMTKGFKRVDESLEDLSLDV-PDAAKKF

SNCLERAKLEGFL

>Aristolochia_elegans_PAWA2930

EYKKKAIVIVEEYFETDDVDFAANEMRYLGHHPY-FYFVKKLVSIAMDRHDKEKEMAAVL

LSSLYSDVIEPQQLYKGFVTLVESADDLSVDIPDTIDVLALFIARAVVDDILPPAFLTKQ

ITALPEGSKGIDTIKRAEKVYLSAPLKAETIERKWGGSKNR-SVEDVKVKINDFLVEYLA

-SGDKIEACRCIKELKLPFFHHEVVKRALSIAMEKRL-AEEPILDLLKEAAEEGVINSSQ

MTKGFERLIDRIDDLSLDIIGARVLLQSLISKAASEGWLCASQLKPFSPKP---------

-------------------------------RKE-AE-EDATKIFKTQITSSIKEYFLTG

DILEMVSSLESENDCNSPELYSIFIKRLITLAMDRKNREKEMASVLLTSLC---LPPEDV

VNGFLLLIESAEDTALDNPVVVDDLAMFLARAVVDEVLAPQHLEEIGSH---CVDPN-TV

GSKAHKMAQSLLQARLSGERILRCWGGG--GSSRPGWAL-EDVKDKITKLLEEYDSGGDV

KEAFRCIKELSMPFFHHEVVKKALVLVMENK-----------------------------

-----------NER-LWGLLDECFSVGLITTSQMTKGFGRVAE---DLVLDI-PDVESQL

DHYVGRAKAKGWL

>Solanum_lycopersicum_XP_004237843

EYKKKAIILVEEYFQNDDITSTANELRELGMSCYDFYFIKKLVSMAMDRHDKEKEMAAVL

LSALYAEVIKPQQVYKGFSKLLESADDFIVDIPDAIDILALFIARAVVDDILPPAFLAKA

NSTLPKDSKGIEVIKRAEKSYLSAPLHAEIIERRWGGSKNK-TVEGVKDKINNLLIEYVV

-SGEKNEACRCINDLNMRFFHHEIVKRAIIMAMEKQQ-AESRLLDLLKKTTEEGLINSSQ

LSKGFNRIIDNIDDLSLDIPNARMIFQSIISKGASEGWLCISSLKSFSTQL---------

-------------------------------EKQEID-EKLVKEFKLKAQSMIQEYFLSG

DIEEVSRILESENSSCLAELNAIFVKKLITLAMDRKNREKEMASVLLSSVC---FPADDV

VNGFVMLIEAADDTALDIPIVVEDLAMFLARAEVDEVLTPQHMEEIGSQ---FFEPN-SI

GNKVVLMAKSLLKGRLSGERILRCWGGG--GSSTNGWAI-EDVKDKIRKLLEEFESGGDA

KEAYRCIKELGMPFFHHEVVKKSLVIIIEKK-----------------------------

-----------SER-LWGFLKECFSMGLITMYQMTKGFARVAESLDDLALDV-PDAEKQF

KVYVERAEAEGWL

>Eschscholzia_californica_EVOD8494

EYKKKVTVIVEEYFATDDVISTANELRELGMPEYSYYFVKKLLSVAMDRHDKEKEMAAVL

VSSLYSHVIEPPQVYKGFSKLVESADDLIVDIPDAVDVLALFIARAVVDDILPPAFLIKE

MGSLPKDSKGVEVLKRAEKSYLSAPLHAEIIERKWGGSKNK-TVEDVKTKINNLLIEYIA

-SGEKIEAFRCIKNLKVPFFHHEIVKRALIIAMEKRE-SEARLLDLLKDATEEGIINSSQ

MTKGFGRLIDTVDDLSLDILSARELLQSLISKAASEGWLCASSLKSLSFQQ---------

-------------------------------QK-QIE-DSAARAFKIKIQYIIQEYFLSG

DILEVISSLESENTTSSSE-----VKKLITTAMDRKSREKEMASVLLSSLS---FPSDSV

INGFVMLVESADDIALDIPAVVEDLAMFLARAVVDEVLAPQHLEEIGSQ---CTGQD-SI

GSKVLQMARSLLRARLSGERILRCWGGG--GSSKPGWEI-DDVKDKIGNALEEYDSGGEL

REAFRCIRELGMPFFHHEVIKKALVTVIEKK-----------------------------

-----------NER-LWGLLEQGFSVGLFTPNQMTKGFGRVADSLDDLALDV-PDAEKQF

SHYVERAKDEGWL

>Medicago_truncatula_XP_003619027

EYKKKATIIVEEYFATDDVVATMSEVREIGKPEYSYYFVKKLVSMSMDRHDKEKEMAAIL

LSALYADIIHPSQVYKGFTKLVESADDLIVDIPDTVDILALFIARAVVDDILPPAFLKKQ

IANLPNDSKGAEVLKKAEKSYLTAPLHAEIIERRWGGSKNT-TVDDVKARINNFLKEYVV

-SGDKTEAFRCIKDLNVPFFHHEIVKRALIMAMEKRQ-AETPLLDLLKEAAEKGFINTSQ

MSKGFTRLIETVDDLSLDIPNARGILQQLMSKAASDGWLCVSSLKPLSIEP---------

-------------------------------EKNTIQ-ENVAKSFKMKTQSIIQEYFLSG

DIFEVISCLEQENNKNCGELNAIFVKKLITLAMDRKNREKEMASVLLSSLC---FPPDDV

VNGFVMLIESADDTALDNPVVVEDLAMFLARSVVDEVLAPQQLEDIGTQ---CISQD-SI

GSKVLQMAKSLLKARLAGERILRCWGGGGGGSSKPGWEI-EDVKDMIGKLLEEYESGGDI

KEACRCMKELGMPFFHHEVVKKSLVKIIEK------------------------------

-----------NER-LWGLLKECFESGLITMYQMVKGFGRVEEALDDLALDV-PDAKNQF

AYYVEKAKNEGWL

>Glycine_max_XP_003548962

NYKKKATIIVEEYFSTDGVIATMNEVKELGKPQYGYYFVKKLVSMSMDRHDKEKEMAAIL

LSALYSDVVDPSQVYKGFSKLVESADDLIVDIPDTVEILALFIARAVVDDILPPAFLKKQ

MAYLPKDSKGVEVLKKTEKSYLAAPLHAEIIERCWGRSKNT-TVDDVKVKINNFLKEYVA

-SGDKKEASRCIKDLKVPFFHHEIVKRVLIMAMERRQ-AESPLLDLLKAAAEEGFINSSQ

MSKGFSRLIDTVDDLSLDIPNARGILQQLMSKAASEGWLCVSSLKSLSEEP---------

-------------------------------EKNTIE-DGAAKSFKVKTQSIIQEYFLSG

DILEVNSCLEQANSKNCAALNAIFVKKLITLAMDRKNREKEMASVLLSSLC---FPADDV

VSGFVMLIESADDTALDNPVVVEDLAMFLARAVVDEVLAPQHLEEIGTQ---CLGPG-SV

GSKVLRMTKSLLKARLAGERILRCWGGG--GSSRSGWAF-EDVKDMIGKLLEEYESGGEI

REACRCMKELGMPFFHHEVVKKALVTTIEKK-----------------------------

-----------NER-LWGLLKECFESGLITMNQMVKGFGRVAESLDDLALDV-PDAKIQF

ANYVERAKANGWL

>Populus_trichocarpa_XP_002307530

EFKKNVTVIVEEYFATDDIVSTANELRELEMSGYHYYFVKKLVSMAMDRDDKEKEMAAVL

LSALYADIIDPQQVYRGFCKLVESADDLIVDIPETVDVLALFIARAVVDDMLPPAFLKKQ

MASLPEDSKGVAVLKRAEKGYLSAPHHAEIIERRWGGGMKK-TVEDVKAKIDNLLQEYAV

-SGDRKEACRCIKDLKVPFFHHEIVKRSLIMAMERKQ-AEGRLLDLLKEASEEGLINSSQ

TSKGFGRMIDSVDDLSLDIPNARRILQSLISKAASEGWLCASSLKSLGPTP---------

-------------------------------VKGSLQ-DDSAKIFKLKAQSIIQEYFLSG

DISEVGSCLGSENNAYSAELNAIFIKRLITLAMDRKNREKEMASVLLSSLC---FPSDDV

VNGFIMLIESADDTALDNPVVVEDLAMFLARAVVDEVLAPRQLEEIGTQ---FSGPE-SI

GRKVLQMAKSSLKARLSGERILRCWGGG--RTGSPGWDI-EDVKDKVGRLLEEFESGGDI

GEACRCIKELSMPFFHHEVVKKALVAIIEKK-----------------------------

-----------NER-LWGLLDQCFSSGLITTCQMMKGFGRVAESLDDLALDV-PDAEKQF

KHYVERATLAGWL

>Ricinus_communis_XP_002527108

EYKKKVTVIVEEYFATDDVVSTANELRELGVPSYNYYFIKKLVSMSMDRHDKEKEMAAIL

ISALYADIIDPSQVYEGFTKLVESADDLIVDIPDTVDILALFIARAVVDDILPPAFIKKE

MASLPADSKGIDVLKRAEKSYLAAPLHAEIIERRWGGSKNK-TVEDVKAKINNLLVECIV

-SGDKKEACRCIKDLKVPFFHHEIIKRALVMAMERQQ-AEGQLLELLKDAAEKGFINTSQ

ITKGFNRVIDAVDDLSLDIPNARGILQSLISKAASEGWLCASSLKSLSVAP---------

-------------------------------VTQPLQ-DSAAKIFKAKAQSIVQEYFLSG

DMSEVSSCLECENSNSSPELNATFVKRLITLAMDRKNREKEMASVLLSSLC---FPADDV

VNGFAMLIESADDTALDNPVVVEDLAMFLARAVVDEVLAPQHLEEIGSQ---FLGLE-SI

GSKVLQMAKSLLKARLSGERILRCWGGA--GSSRPGWAV-EDVKDKIGKLLEEFESGGDI

REAYRCIKELGMPFFHHEVVKKALVTIIEKK-----------------------------

-----------SRR-LWGLLEESFHSGLITSYQMMKGFGRVAESLDDLALDV-PDAEKQF

VQYVEKAKIAGWL

>Vitis_vinifera_XP_002264439

EYKKKAAVIVEEYFATDDVVSTASELREISLPRYNFYFVKKLVSMAMDRHDKEKEMAAVL

LSALYADVIDPSQVYKGFGKLVESSDDLIVDIPDTIDVLALFVARAVVDDILPPAFLTKH

LASLPKDSKGVQVLRRAEKGYLAAPLHAEIIERRWGGSKNT-TVEDVKARINNLLVEYRV
-SGDVKEACRCIKDLKVPFFHHEIIKRALIMAMERRH-AEDRLLDLLKAAAEEGLINSSQ
ISKGFGRMIDSVDDLSLDIPSAKSILKSLISKAASEGWLSASSLKSLSLEP---------
--------------------------------EKRSLE-DNVARTFKLKAQSIIQEYFFSG
DISEVSSCLESENSPSSAELNAIFVKRLITLAMDRKNREKEMASILLSSLC---FPADDV
VNGFVMLIESADDTALDIPVVVEDLAMFLARAVVDEVLAPQHLEEIGSQ---CLSPD-SI
GSKVLQMAKSLLKARLSGERILRCWGGG--GSGSTARAV-EDVKDKIGKLLEEYESGGDF
REACRCIKELGMPFFHHEVVKKALVTVIEKK------------------------------
-----------NER-LWRLLRECFGSGLITMYQMMKGFSRVGEALDDLALDV-PDAKKQF
TYYVEQAKIAGWL
>Gossypium_raimondii_Gorai005G0764001
AFKKKATIIVEEYFATDDVDSATNELKELAMPSYNYYFVKKLVSMAMDRHDHEKEMAAVL
LSALYADVIDAPQVYRGFSKLVESADDLIVDIPDTVDILALFIARAVVDDILPPAFLKKQ
IALLPNDSKGVEVLERAEKGYLAAPMHAEIIERRWRVSK-K-TVEDVKARINNLLIEYVT
-SGDKKEAYRCIKDLKVPFFHHEIVKRALVMAMERRQ-AEDRLLDLLKEVAEEGLINSSQ
ITKGFDRMIDTIEDLSLDIPNAQRILKSLISKATSDGWLCASSLRSLSLEP---------
--------------------------------RKKLLE-DNFTRTFKLKSQSIVQEYFLTG
DISEVFSCLEAENRTSSGELNAIFIKRLITLAMDRKNREKEMASVLLSSLC---FPADDV
VNGFVMLIESADDTALDNPVVVEDLAMFLARAVVDEVLAPQHLEEVGSQ---FLGTD-SI
GGKVLQMAKSLLKARLSGERILRCWGGG--GSSRPGWAV-EDVKHKIGKLLEEYESGGDI
REAYRCIKELGMPFFHHEVVKKAMVMVMEKK------------------------------
-----------NDR-LWGLLAHCFGSGLITMNQMTKGFSRVEESLDDLALDV-PDARKQF
LAYVEKAKTTGWL
>Theobroma_cacao_Thecc1EG039086t1
AYKKKTTIIVEEYFATDDVVSTTNELRELAMPSYNYYFVKKLVSMAMDRHDQEKEMAAVL
LSALYADVIDAPQVYKGFSKLVESADDLIVDIPDTVDVLALFIARAVVDDVLPPAFLKKQ
IAFLPNGSKGLEVLKRAEKGYLAAPMHAESIERRWGGSKTK-TVEDVKARINNLLIEYVV
-SGDKKEAFRCIKDLKVSFFHHEIVKRALIMAMERHQ-VEDRILDLLKEATEEGLINSSQ
ITKGFDRMIDTVDDLSLDIPNAQRILKSLISNAASEGWLCASSLKSLSLEP---------
--------------------------------KKKLLE-DSFTRTFKIKSQSIIQEYFLSG

DISEVCSCLEVENKTSSGELNAIFIKRLITLAMDRKNREKEMASVLLSSLC---FPVDDV

VNGFAMLIESADDTALDNPVVVEDLAMFLARAVVDEVLAPQHLEEVGSQ---FLGTD-ST

GSRVLQMAKSLLKARLSGERILRCWGGG--GSSRPGWAV-EDVKDKIGKLLEEYESGGDV

REACRCIKELGMPFFHHEVVKKALVTVMEKK----------------------------

-----------NER-LWGLLRHCFGSGLITMNQMTKGFVRVAESLDDVALDV-PDAQKQF

LNYVERAKTKGWL

>Chaetosphaeridium_globosum_HO379087

--VKAKIQELLVELLS

-SGDVAEACRCIRELNMPFFHHEVVKRALTLAVEKD--GSGPVLKLLADCAESGLITSNQ

MRKGFARAVDAVEDLTLDVPHAREALISTVTEAAKEGWLDERFATVALAELE--------

--------------------------NSPRAEMESPLDSPAAAEFKEHATRVIQEYFSSG

DIGEVARSIEEL--APPPEFHHLFVKRLITLAMDRKVREHEMASVLLSVLAGDTLSPVHI

GQAFSRLLEALEDLALDIPDASTELALFLERLVVDDVLAPLYVSEMAEE----LPKGSL-

GSETVHMVQAVLSARHAGERVLRCWGGG------TAVTV-EEAKDRMGKLLEEFEAGGEL

AEACQCIRDLDMPFFHHELVKQALILAMEKQ-----------------------------

-----------NFL-VLELLKECANEALVTTSQMSLGFGRVFDRLDDLALDN-PRARQVY

DSYVSLGQEEGWL

>Selaginella_moellendorffii_XP_002979104

EFKGRVLLAIEEYFMSSNIDEAAQELRDLGCPDFQHYFVKKLVSIAMDKRDREKEKAAVL

LSALYADVVPADQMAKGFRKLLLSVDDLALDNPNAVKILAVFVARAVVDDILPPAFLTDA

QKLLAEGSKGMEVVNKAMATHLGPSAHADMVEKKWGGSTRS-TVALLVKKIDEMLEEYRE

-SGDVTKACQCIRELDMGYYHHELVKRAATLSLEGNKSTQSSLLALLKHCSEEGLISSSQ

MSKGFMRCLEES------SAEAREKLKPVVSAAVNEGWLSPSLQTSLASA----------

----------------------------------APEPDFSSAEFKKKSTAIIHEYFSSD

DSQEVLRSLQDL--ASVQDLYPLFIKRLILLAMDRRSREKEMASSLLSTIHTES-DTDQV

AKGFVLLLESAEDTALDTPDAGTQLTFFLARAVFDNVLTPFYLEQIKGQ----LPENSL-

GREIVGNAKSILSAQHAGERILRCWGGG------TGWAI-EDAKDKVFKIVEEFEAGGDL

TEACRCIRELNMPFFHHEIVKRVLDMAMEKQ-----------------------------

-----------NER-PLELLEQCSREGLITTSQMCAGFTRVYNLLNELALDV-PNAHEKF

QSYVETAKQAKWL

>Sorghum_bicolor_XP_002448072

DYRKLVVPIIEEYFSTGDVELAASELRGLGSDQFQHYFVKKLISMAMDRHDKEKEMASIL

LSSLYADLLSSYTISQGFMMLLESTEDLTVDIPDATDVLAVFIARAIVDEILPPIFLTRA

RALLPEFSKGIQVLQVVEKSYLSAPHHAELVERKWGGSTHF-TVEEAKRRIQNILREYIE

-SGDIDEAFRCIRELSLPFFHHEVVKRALTFAMENI-SSQPLILKLLKEAAAGCLISPNQ

ISKGFSRLAEGVDDLSLDIPSAKALFDKLVSTAMAEGWLDASFGKSAAPD----------

-------------------------------EEMQNTSAVKMKHFKEESAHIIHEYFLSD

DVPELIRSLEEL--SA-PEFNPIFLKKLVTLAMDRKSREKEMASVLLSSLRLELFSTEDI

MKGFIMLLQSAEDTALDIVDAPSELALFLARAVIDEVLIPLNLDEISIK----LRPNSS-

GSQTVQMARALLSARHSGERILRCWGGG------TGWAV-EDAKDKITKLLEEYNTGGDL

GEACRCIRDLGMPFFNHEVVKKALVMAMEKQ-----------------------------

-----------NDTSILALLQECFGEGLITINQMTKGFARVKEGLDDLVLDI-PNAQEKF

GEYVELATEHGWL

>Zea_mays_NP_001147914

EYRKLVVPIIEEYFSTGDVELAASELKCLGSDQFHHYFVKKLISMAMDRHDKEKEMASIL

LSSLYADLLSSYRISEGFMMLLESTEDLTVDIPDATDVLAVFIARAIVDEILPPVFLTRA

RALLPEFSKGIQVLQVVEKSYLSAPHHAELVERKWGGSTHS-IVEEAKSKIKNILREYIE

-NEDIDEAFRCIRELSLPFFHHEVVKRALTFGIENV-SSQPSILKLLKEAAASCLISPNQ

ISKGFSRLAEGVDDLSLDIPSAKVLFDKLVSTAISEGWLDASFGKSAAPD----------

-------------------------------EEIQSTSVVKVKRFKEECGHIIHEYFLSD

DVPELIRSLEEL--SA-PEFNPIFLKKLVTLAMDRKSREKEMASVLLSSLRLELFSTDDI

IEGFVMLLQSAEDTALDIVDAPSELALFLARAVIDEVLIPLNLDEISIK----LRPNSS-

GSQTVQMARALLSARHSGERILRCWGGG------TGWAV-EDTKDKITKLLEEYNTGGDL

GEACRCIRDLGMPFFNHEVVKKALVMAMEKQ-----------------------------

-----------NDTSILVLLQECFGEGLITINQMTKGFARVKEGLDDLILDI-PNAQEKF

GEYVELATERGWL

>Brachypodium_distachyon_XP_003579994

DYRKLVVPVIEEYFSTGDVSLVASELKSLGSDQFHNYFVKKLISMAMDRHDKEKEMASIL

LSALYADLLGSSKMSEGFMMLLESAEDLSVDIPDAVDVLAVFIARAVVDEILPPVFLARA
RALLPESSKGIEVLLVAEKSYLSAPHHAELVERKWGGSTYF-SVEEAKKRIQDILREYVE
-SGDANEAFRCIRELGLPFFHHEVVKRALILGMENL-SSQPLILKLLKESAAGCLITSNQ
VSKGFARVAESVDDLSLDIPSAKTLFDKLLSAAISEGWLDASFTKSAASD----------
------------------------------EDMVTASGEKVKRFKVESGHIIQEYFLSD
DIPELIRSLQEL--SA-PEYNAIFLKKLITLAMDRKNREKEMASVLLSSLRLELFSTDDI
MKGFIMLLQSAEDTALDIVDAPSELALFLARAVIDEVLIPLNLDDISIK----LRPNSC-
GSQTVQMASALLAARHSGERILRCWGGG------TGWAV-EDAKDKISKLLEEYKTGGDL
GEACQCIRDLGMPFFNHEVVKKALVMAMEKQ----------------------------
-----------NEASILALLQECFSEGLITINQMTNGFGRVKEGLDDLILDI-PNAQEKF
GAYVELATGRGWL

>Oryza_sativa_OSJNBb0011N17

DYKRLLVPVIEEYFSTGDVELAASELRSLGSDQFHSYFIKKLISMAMDRHDKEKEMASIL
LSALYADLLGSSKMSEGFMMLLESTEDLSVDIPDAIDVLSVFVARAVVDEILPPVFLTRA
RALLPEFSKGIEVLQVSEKSYLSAPHHAELVERKWGGSTHF-TVEEAKRRIQDILKEYIE
-SGDIDEAFRCIRELGLPFFHHEVVKRALTLSMENL-SSQPLILKLLKESTAGCLISSNQ
MSKGFCRLAESIDDLSLDIPSAKILFDKLVLTATSEGWLDASFTTSSAPN----------
------------------------------EDMRNASGEKIKHFKEESGHIIQEYFLSD
DVPELIISLQEL--SA-PEYNPIFLKKLITLAMDRKNREKEMASALLSSLSLELFSTDDI
MKGFILLLQSAEDTALDIVDAPSELALFLARAVIDEVLIPLNLDEIGNR----LRPNSS-
GSQTVQMARALLAARHSGERILRCWGGG------TGWAV-EDAKDKIAKLLEEYNTGGDL
GEACQCIRDLGMPFFNHEVVKKALVMAMEKE----------------------------
-----------NEARILALLQECFGEGLITINQMTLGFTRVKEGLDDLILDI-PNAQEKF
GAYVDLATERGWL

>Arabidopsis_thaliana_MAT6

DYKKSVVSIIDEYFSTGDVKVAASDLRELGSSEYHPYFTKRLVSMAMDRHDKEKEMASVL
LSALYADVILPDQIRDGFIRLLRSVDDLAVDILDAVNVLALFIARAIVDEILPPVFLVRS
KKILPESCKGFQVIVTAEKSYLSAPHHAELVEKKWGGSTHT-TVEETKKKISEILKEYVE
-NGDTYEACRCIRELGVSFFHHEVVKRALVLAMDSP-TAESLVLKLLKETAEEGLISSSQ
MVKGFFRVAESLDDLALDIPSAKKLFDSIVPKAISGGWLDDSFKIT-SDQ----------

-------------------------------DGEKSSQDGKLRQYKKDTVNIIQEYFLSD

DIPELIRSLQDL--GA-PEYNPVFLKRLITLALDRKNREKEMASVLLSALHMELFSTEDF

INGFIMLLESAEDTALDIMDASNELALFLARAVIDDVLAPLNLEDISTK----LPPKST-

GTETVRSARSLISARHAGERLLRSWGGG------TGWIV-EDAKDKISKLLEEYETGGVT

SEACQCIRDLGMPFFNHEVVKKALVMAMEKQ-----------------------------

-----------NDR-LLNLLEECFGEGLITTNQMTKGFGRVNDSLDDLSLDI-PNAKEKF

ELYASHAMDNGWI

>Capsella_rubella_Carubv10027802m

DYKKAVVSIIEEYFSTGDVKVAASDLRELGSSEYHPYFTKRLVSMAMDRHDKEKEMASVL

LSALYADVILPDQIRDGFIRLLRSVDDLAVDILDAVNVLALFIARAIVDEILPPIFLARS

KKILPESSKGFQVIVTAEKSYLSAPHHAELVEKKWGGSTHT-TVEETKKKISEILKEYVE

-NGDTYEACRCIRELGVSFFHHEVVKRALVLAMECQ-TAESLLLKLLNEAAEEGLISSSQ

MVKGFYRVAESLDDLALDIPSAKKLFDSIVPKAISGGWLDESFKVVTSVK----------

-------------------------------DGGKSSEEGKLRQYKKDTVNIIQEYFLSD

DIPELIRSLQDL--GT-PEYNPVFLKRLITLALDRKNREKEMASVLLSALHMELFSTEDF

INGFIMLLESAEDTALDIMDASNELALFLARAVIDDVLAPLNLEEISTK----LPPKST-

GTETVRSARSLISARHAGERLLRSWGGG------TGWIV-EDAKDKILKLLEEYETGGVT

SEACQCIRDLGMPFFNHEVVKKALVMAMEKQ-----------------------------

-----------NDR-LLNLLEECFGEGLITTNQMTKGFGRVKDSLDDLSLDI-PNAKEKF

KLYASHAMDNGWI

>Thellungiella_halophila_Thhalv10003735m

DYKKAVVSIIEEYFSTGDVNVAASDLRELGSSEYHPYFTKRLVSLAMDRHDKEKEMASVL

LSALYADVISPDQIRDGFIRLLRSVDDLVVDIPDAVNVLALFIARAIVDEILPPVFLSRS

KKSLPESSKGFQVIVTAEKSYLSAPHHAELVERKWGGSTHT-TVEETKKKISEILKEYVG

-NGDTYEACRCIRELGVSFFHHEVVKRALVLAMESQ-TAESRILKLLNEAAEEGLISSSQ

MMKGFYRVAESLDDLALDVPSAKKLFDSIVPKAISGGWLDDSFKVT-SDL----------

-------------------------------GGGKSSQDEKLRQYKKDTVNIIQEYFLSD

DIPELIRSLEDL--AT-PEYNPVFLKKLITLALDRKNREKEMASVLLSSLHMELFSTEDF

INGFIMLLESAEDTALDIMDASNELALFLARAVIDDVLAPLNLEEISIK----LPPKST-

GTETVRSARSLISARHAGERLLRSWGGG------TGWIV-EDAKDKILKLLEEYETGGVT

SEACQCIRDLGMPFFNHEVVKKALVMAMEKK----------------------------

-----------NDG-LLNLLEECFGEGLITSNQMTKGFGRVKDSLDDLSLDI-PNAKEKF

ELYVGHAMDNGWI

>Brassica_rapa_Bra038615

DYKKAVVSIIEEYFTTGDVKVAASDLRELGSSEYHPYFTKRLVSMAMDRHDKEKEMASVL

LSALYADVILPDQIRDGFIRLLRSVDDLAVDIPDAVNVLALFIARAIVDEILPPVFLARS

KKNLPESSKGFQVIVTAEKSYLSAPHHAELVERKWGGSTHT-TVEETKKKISEILKEYVE

-NGDTYEACRCIRELGVTFFHHEVVKRALVLAMESQ-AAESLILKLLNEAAEEGLISSSQ

MVKGFNRVAESLDDLALDIPSARKLFDSIVPKAISGGWLDDSFKVN-PDQ----------

-------------------------------DGRESSQDDKLRQYKKDTVNIIQEYFLSD

DIPELIRSLEDL--AT-PEYNPVFLKKLITLALDKKNREKEMASVLLSSLHMELFSTEDF

INGFIMLLESAEDTALDIMDASNELALFLARAVIDDVLAPLNLEEISTK----LPPKST-

GTETVRSARSLISARHAGERLLRSWGGG------SGWIV-EDAKDKILKLLEEYEMGGVT

SEACQCIRDLGMPFFNHEVVKKALVMAMEKK----------------------------

-----------NDG-LLNLLEECFGEGLITMNQMTKGFGRVKDSLDDLSLDI-PNAKEKF

ELYVGRAMDNGWI

>Arabidopsis_thaliana_MAT7

DYKREVVSIIDEYFSSGDVEVAASDLMDLGLSEYHPYFVKRLVSMAMDRGNKEKEKASVL

LSRLYALVVSPDQIRVGFIRLLESVGDLALDIPDAVNVLALFIARAIVDEILPPVFLARA

KKTLPHSSQGFQVILVSENSYLSAPHHAELVETKWGGSTHI-TVEETKRKISEFLNEYVE

-NGDTREACRCIRELGVSFFHHEIVKSGLVLVMESR-TSEPLILKLLKEATEEGLISSSQ

MAKGFSRVADSLDDLSLDIPSAKTLFESIVPKAIIGGWLDEDSFK---ER----------

-------------------------------SD-QNGGSENLRRFKKDAETIIQEYFLSD

DIPELIRSLEDL--GL-PEYNPVFLKKLITLAMDRKNKEKEMASVFLASLHMEMFSTEDF

INGFIMLLESAEDTALDILAASDELALFLARAVIDDVLAPLNLEEISNS----LPPKST-

GSETIRSARSLISARHAGERLLRSWGGG------TGWAV-EDAKDKIWKLLEEYEVGGVI

SEACRCIRDLGMPFFNHEVVKKALVMAMEKK----------------------------

-----------NDR-MLNLLQECFAEGIITTNQMTKGFGRVKDSLDDLSLDI-PNAEEKF

NSYVAHAEENGWL

>Capsella_rubella_Carubv10016789m

DYKRSVVSIIEEYFISVDVQVAASDLMELGLSEYHPYFVKRLVSMAMDRGHREKEKASVL
LSRLYPNIVSPDQIRVGFIRLLESIGDLALDIPDAVNVLALFISRAIVDEILPPVFLARA
KKTLPDSSQGFQVILTAEKSYLSAPHHTELVEKKWGGSTCI-TVEETKRKISEILKEYVE
-NSDTREACRCIRELGISFFHHEVVKRALVLAMEFQ-TSEPLLLKLLKEAAEAGLISSSQ
MAKGFSRVAESLDDLSLDIPSAKTLFESIAPKAVSGGWLTLKECN---DQ----------
-------------------------------NESLPIEDEKLRQYKKDTVIVIQEYFLSD
DIPEVIRSLEDL--GS-PEYNPVFLKKLITLAMDRKNKEKEMTSVLLPALHMEMFSTEDF
INGFIMLLESAEDTALDILEASHELALFLARAVIDDVLAPLDLEDISNF----LPLKSA-
GNETIRSARSLITATHAGERLLRCWGGG------TGQGV-EDAKDKIWKLLEEYETGGVI
LEACQCIRDLGMPFFNHEVVKKTLVMAMEKK-----------------------------
-----------NDM-MLNLLQECFAEGIITTNQMTKGFGRIKDSLDDLSLDI-PNAEEKF
NSYVSHAEENGWL

>Sorghum_bicolor_XP_002444968

DYKKAVIPLIEEYFSNGDVKLAASDLKELGYDDFHRYFVKKLVSTAMDRHDKEKEMASVL
LSYLYGNVVSSTQIRLGFVLLLEAVDDLAVDIPDVVDVLALFVARAVVDDILPPAFLSKA
KVSLSGSSKGMQVVQIAEKSYLSAPHHAELIERRWGGSTHI-TVEEVKKRIADLLKEYIR
-NGDTAEACRCIRELAVPFFHHEVVKRALTLGMESP-AAEALIAKLLKEASEECLISSSQ
MMKGFYRVAESLDDLILDIPSAKSEFQLLVSKAISEGWLDSSYV-KSGVN----------
-------------------------------GSVEDDEHEKLARYKREAVSIIHEYFLSD
DTTEVIRSVKEL--GY-PEYNPIFIKKLITTAMDRKNREKEMTSVLLSSLSMELFSSEDI
AKGFIMLLESAEDTALDILDASDELGLFLARAVIDDVLAPLNLDEISSK----LPPNCS-
GAETLNMARSLVSARHAGERLLRCWGGG------TGWAV-EDAKDKITKLLEEYESGGDL
GEACNCIRELGMSFFNHEVVKKALVMAMEKK-----------------------------
-----------NER-TLSLLQECFGEGIITINQMTKGFSRVRDGLDDLALDI-PDAREKF
ISYVEHAKKSGWL

>Oryza_sativa_Os08g0120500

DYKKSVAPIIEEYFSTGDVKLAASDLKELGYDDFHRYFVKKLVSMAMDRHDKEKEMASVL
LSSLYGDVISSTQIRLGFVMLLEAVDDLAVDILDAVDVLALFIARAVVDDILPPAFLSRE
KASLSESSKGMQVVQIAEKSYLSAPHHAELLERRWGGSTRT-TVDAVKLRITDLLKEYIK
-NGDTAEACRCIRELAVPFFHHEVVKRALTLGMESP-TAEALIVKLLKEASEELLISSSQ

MMKGFSRVVDSLDDLSLDIPSAKSQFQTLVSKAVSEGWLDSSFV-HVGAN----------
-------------------------------GDVQDDEHEKLRRYKKEAVSMIHEYFLSD
DVPEIIRSLKEL--GS-PEYNPVFIKKLITIAMDRKNREKEMASILLSSLSMELFSTEDI
VKGFIMLLESAEDTALDILDASDELGLFLARAVIDDVLAPLNLDEISGK----LPPNCS-
GAETLNMARSLATARHAGERLLRCWGGG------TGWVV-EDTKDKIAKLLEEYESGGDV
GEACNCIRELHMPFFNHEVVKKALVMAMEKK-----------------------------
-----------NDR-ILGLLQECFGEGIITINQMTKGFSRVRDGLDDLALDI-PDAREKF
LSYVEHAKKSGWL
>Hordeum_vulgare_BAJ94459
DYKKSLVMIIEEYFSTGDVKQTGSDLKELGYDDFHRYFVKKLVSMAMDRHDKEKEMVSVL
LSCLYGDGLSSTQIKLGFVMLLQAVDDLAVDIPDAVDVLALFIARAVVDDILPPAFLNKA
KGSLTEASKGMQVLQIAAKSYLSAPHHAELLERRWGGSTHI-TVEEVKRRITDLLKEYIK
-NGDTAEACRCIRELAVPFFHHEVVKRAVTLGMESP-AAETLIAKLLKEASEEGLISSSQ
MAKGFSRIVESLDDLSLDIPSAKSQFQTLVSKAVSEGWLDSSYE-PSGAN----------
-------------------------------GNVQDDDHEKLRKYKREAVSMIHEYFLSD
DIAELIRTLEEL--GL-PEYNPVFIKKLITIAMDRKNREKEMASVLLSSLSMELFSSEDI
VKGFIMLLESAEDTALDILDASDELGLFLARAVIDDVLAPLNLDEIGSI----LPANCS-
GAETLNMARSLASARHAGERLLRCWGGG------TGWAV-EDAKDKITKLLEEYESGGDV
GEACKCIREMGMPFFNHEVVKKALVMAMEKK-----------------------------
-----------KEG-TLALLHECFGEGIITINQMTKGFSRVRDGLDDLALDI-PDARDKF
LSYVENGKKNGWL
>Brachypodium_distachyon_XP_003573315
DYKKSLVTIIEEYFSTGDVKLASSDLKELGHDDFHRYFIKKLVSMAMDRHDKEKEMVSVM
LSSLYGDVFSSTQIKLGFVMLLQAVDDLTVDIPDAVDVLALFIARAVVDDILPPAFLSKA
KGSLSESSKGMQVLQIAAKSYLSAPHHAELLERRWGGSTYI-TVEEVKRRITDLVKEYIK
-NGDTTEACRCIRELAVPFFHHEVVKRAVTLGMESP-ATEALIIKLLKEAAEEGLISSSQ
MMKGFSRVAESLDDLSLDVPSAKSQFQTLVPKAVSEGWLDSSYV-PSGAN----------
-------------------------------GHVQDDDHEKLRRYKKEAVSMIHEYFLSD
DIPELIRTLKEL--GF-PEYNPIFIKKLITIAMDRKNREKEMASVLLSSLSMELFSTEDV
VKGFIMLLESAEDTALDILDASDELGLFLARAVIDDVLAPLNLDEIGSE----LPPKCS-

GAETLNMARSLASARHAGERLLRCWGGG------TGCAV-EDAKDKIAKLLEEYESGGDL

GEACNCIREMGMPFFNHEVVKKALVMAMEKK-----------------------------

-----------TER-TLELLQECFGEGIITINQMTKGFSRVRDGLDDLALDI-PDAPEKF

LSYVEHAKKNGWL

>Arabidopsis_thaliana_MAT5

DYKKAAASIINEYFSTGDVDVAAADLIELGSSEYHPYFIKRLVSVAMDRHDKEKEMASVL

LSALYADVINPNQIRDGFVLLLESADDFVVDIPDAVNVLALFLARAVVDDILPPAFLPRA

AKALPITSKGYQVVQTAEKSYLSAAHHAELVERRWGGQTRT-TVEEVKKKIADILNEYVE

-TGETYEACRCVRELGVSFFHHEVVKRALVTALENH-AAEAPVLKLLNEAASENLISSSQ

MVKGFSRLRESLDDLALDIPSARTKFGLIVPKAVSGGWLDASFGYPSGEC----------

-------------------------------GRQQNE-DEKLKRFKEDIVTIIHEYFNSD

DIPELIRSLEDL--GA-PEYNPIFLKKLITLALDRKNHEKEMASVLLSSLHIEMFTTEDV

ADGFVMLLESAEDTALDILDASNELALFLARAVIDDVLAPFNLEEISSK----LRPNSS-

GTETVKMARSLIFARHAGERLLRCWGGG------SGWAV-EDAKDKISNLLEEYESSGLV

SEACKCIHELGMPFFNHEVVKKALVMGMEKK-----------------------------

-----------KDKMMLDLLQESFSEGLITTNQMTKGFTRVKDGLEDLALDI-PNAKEKF

NDYVEYGKKNGWV

>Capsella_rubella_Carubv10007684m

DYKKAVASIIEEYFSTGDVDVAAADLIELGSSEYHPYFIKRLVSVSMDRHDKEKEMASVL

LSALYADVINPNQIRDGFVLLLESADDFVVDIPDAVNVIALFLARAVVDEILPPAFLPRA

AKALPVTSKGYQVVQTAEKSYLSAAHHAELVERRWGGQTRT-TVEEVKKKISDILKEYVE

-TGETYEACHCVRELGVSFFHHEVVKRALVIALENN-AAEAPVLKLLKEAAAENLISSSQ

MVKGFSRLRESLDDLALDIPSAKTKFDLIVPKAISGGWLDASFSYPSGEC----------

-------------------------------GRQQVE-DEKLKRFKEEILTIIHEYFNSD

DIPELIRSLEDL--GA-PEYNPIFLKKLITLALDRKNHEKEMASVLLSALHIEMFTTEDV

ADGFVMLLESAEDTALDILDASNELALFLARAVIDDVLAPFNLEEISSK----LRPNSS-

GTETVKMARSLIFARHAGERLLRCWGGG------SGWAV-EDAKDKISNLLEEYESSGLV

SEACKCIRELGMPFFNHEVVKKALVMGMEKK-----------------------------

-----------KDKMMLDLLQESFGEGLVTTNQMTKGFTRVKDGLEDLALDI-PNAKEKF

KDYVEHGKKNGWV

>Thellungiella_halophila_Thhalv10024556m

DYKKAVASIIEEYFSTGDVDVAASDLIELGSSEYHPYFIKRLVSVAMDRHDKEKEMASVL

LSALYADVINPNQIRDGFVLLLESADDFVVDIPDAVNVLALFLARAVVDDVLPPAFLPRA

TKALPQSSKGYQVVQTAEKSYLSAAHHAELVERRWGGTTRT-SVEEVKKKITDILKEYVE

-TGDTYEACRCIRELGVSFFHHEVVKRALITGMESY-TSEPLVLKLLKEAASENLINSSQ

MVKGFSRLRESLDDLALDIPSAKTKFDLLVPKAIAGGWLDASFSYPSGEN----------

-------------------------------GRQQIE-DEKLKRFKEEIVTIIHEYFNSD

DIPELIHSLEDL--GA-PEYNPIFLKKLVTLALDRKNREKEMASVLLSSLHIEMFTTEDV

ADGFVLLLESAEDTALDILDASNELALFLARAVIDDVLAPFNLEEICSK----LRPNSS-

GTETVKMARSLIFARHAGERLLRCWGGG------TGWAV-EDAKDKILNLLEEYESSGLV

SEACKCIRELGMPFFNHEVVKKALVMAMEKK-----------------------------

-----------NDKMVLDLLQECFGEGLITTNQMTKGLTRVKDGLEDLALDI-PNAKEKF

KDYVEHAKKNGWV

>Brassica_rapa_Bra013836

DYKKAVASIIDEYFSTGDVDVAAADLIELGSSEYHPYFIKRLVSVSMDRHDKEKEMASVL

LSSLYTDVINPNHIRDGFVLLLESADDFVVDIPDAVNVLALFLARAVVDDILPPAFLPRA

SKALPASSKGYQVVQTAEKSYLSAAHHAELVERRWGGMTRT-SVEEVKKKIADILKEYME

-TGDAYEACRCIRELGVSFFHHEVVKRALISGMESD-AAEPLVLNLLKEAASENLISSSQ

MVKGFSRLRERLDDLALDIPSAKTKFDLIVPKAISGGWLDASFSAPSGES----------

-------------------------------GRQEME-DEKLKRFKEEVVTIIHEYFNSD

DIPELIRSLEDL--GA-PEYNPIFLKKLVTLALDRKNREKEMASVLLSSLHIEMFTTEDV

ADGFVMLLESAEDTALDILDASNELALFLARAVIDDVLAPYSLEEISSR----LVPNSS-

GTETVKMARSLIFARHAGERLLRCWGGG------TGWAV-EDAKDKILNLLEEYESSGLV

SEACKCIHELGMPFFNHEVVKKALVMAMEKK-----------------------------

-----------KDKIVVELLQESFGEGLITINQMTKGFTRVKDGLEDLALDI-PNAKEKF

NEYVEHAKKNGWV

>Solanum_lyopersicum_LOC101255979

DYKKSVASIIEEYFSTGDVEVATSDLKELGSAEYHPYFIKRLVSMSMDRHDKEKEMASVL

LSALYADVINPTQISQGFFMLVESADDLAVDIPDTVDILALFIARAVVDDILPPAFIARA

RKMLPESSKGIQVLQTAEKSYLSAPHHAELVERRWGGSTHF-TVEEVKKRIADLLREYVE

-SGDTAEACRCIRKLEVSFFYHEVVKRALVLAMEMQ-SAEPLILKLLKEAAEEGLISSSQ
MVKGFSRMAESIDDLSLDIPSAKMSFQSIVPRAISEGWLDATSLKASGED----------
------------------------------GPANGPDDEKVKQYKKQIVNIIHEYFLSD
DIPELIRSLEDL--VA-PEYNPIFLKKLITLAMDRKNKEKEMASVLLSALHIEIFSTEDI
VNGFVMLLESAEDTALDILDASNELALFVARAVIDDVLAPLNLEEITSR----LPPNCSS
GAETVCMAQSLLSARHAGERILRCWGGG------TGWAV-EDAKDKIQKLLEEFESGGVM
SEACQCIRDMGMPFFNHEVVKKALVMAMEKK----------------------------
-----------NDR-MLDLLQECFSEGLITINQMTKGFGRIKDGLDDLALDI-PNAKDKF
MFYVEHAKGNGWV
>Solanum_lyopersicum_LOC101256273
DYKKSVVSIIEEYFSSADVELAASDLKELGSTDYHPYIIKRLVSMAMDRHDKEKEMTSVL
LSSLYADVINPTQIRQGFYMLVESADDLAVDIPDTVDILALFIARAVVDDILPPAFIARV
GKMVPESSKGFQVLQTAEKRYLSAPHHAELVERRWGASTQF-TVEEVKKRIADLLREYVE
-SGDIAEACRCIRQLELPFFYHEVVKRALVLAMEIQ-SAEPLILKLLKEAAEEGLISSSQ
MVKGFSRMAESIDDLSLDIPSAKMSFQSIVPRAISEGWLDASSLIATGEN----------
------------------------------GQANGPDDEKLKQYKKQIVSIIHEYFLSD
DIPELIQSLEDL--GQ-PEFNPIFLKKLITLAMDRKNKEKEMASVLLSALHIEIFSTEDI
VNGFVMLVESSEDTALDILDASNELALFLARSVIDDVLAPLNLEEILNK----LPPNCIS
GAETIRTAQSLRSARHAGERILRCWGGG------TGWAV-EDAKDKIQKLLEEFESSGVL
SEACHCIRELGMPFFNHEVVKKALVMAMEKK----------------------------
-----------NDR-MLDLLQACFSEGLITINQMTKGFGRINDGLEDLALDI-PNAKDKF
TFYLEHAKERGWM
>Eschscholzia_californica_TUHA2426
DYKKAVVSLIEEYFSNGDVELAATDLKDLGSSEYLPYFVKRLVSIAMDRHDKEKEMASVL
LSALYADVISATQISQGFVLLLEAADDLAVDIPDAVDVLALFVARAVVDDILPPAFLSRA
KKVLSESSKGLQVLQIAEKSYLSAPHHAEFVEKRWGGSTHV-TAEEVKNKISVLLREYVG
-SGDTKEACRCIRELGVPFFHHEVVKRALVIAMEIQ-TGQPLVLKLLKEAAEEGLISSSQ
MVKGFGRFAESLDDLSLDIPSAKSLFQSLVPKAISEGWLDSSFLDEN-------------
------------------------------GDAQNVDADKVRSFKEESVTIIHEYFLSD
DIPELIRSLEDL--AA-PEFNPVFLKKLITLAMDRKNREKEMASVLLSALHTEIFSTEDI

VSGFVMLLESAEDTALDILDASNELALFLARAVIDDVLAPLNMEEISSK----LPPNCS-

GAETVHVARSLVAARHAGERILRCWGGG------TGWAV-EDAKDKITKLLEEYESGGVV

SEACQCIRDLGMPFFNHEVVKKALVMAMEKK-----------------------------

-----------NDR-MLDLLQECFGEGLITINQMTKGFSRIRDGLEDLALDI-PNANEKF

SFYVEHAKNHDWL

>Aristolochia_elegans_PAWA5255

DFKKVVVPIIEEYFNTGEVEVAASDLRDLGSFEYHHYFVKRLVSLAMDRHDKEKEMASVL

LSALYADVISSDQISQGFIMLLESVDDLALDILDAVDVLALFIARAVVDDILPPAFLTRA

MKKFPESSKALQVVQTAEKSYLSAPHHAELVERRWGGTTHV-TVEEVKKKIADLLREYVE

-SGDTAEACRCIRELGVSFFHHEVVKRALIVAMEIR-SAEPLVLKLLKEAADEGLISSSQ

MAKGFLRLAESLDDLSLDIPTAKSMFEEMVPVAIAEGWLDASFQKMSGES----------

-------------------------------REV---DVEKVRKYKEEAVTIIHEYFLSD

DIPELIRSLEDL--AA-PEFNPIFLKKLITLAMDRKNREKEMASVLLSSLYTEIFSPDDI

VNGFVLLLESAEDTALDILDASNELALFLARAVIDDILVPLNLEEISSK----LPPNCT-

GTETVHMARSLLSARHAGERILRCWGGG------TGWAV-EDAKDKIIKLLEEYEAGGVV

SEACQCIRDIGMPFFNHEVVKKALIMAMEKK-----------------------------

-----------NDR-MLDLLQECFGEGLITINQMTKGFARIRDGLDDLALDI-PDAEEKF

RFYVEHAKRNGWL

>Medicago_truncatula_XP_003608913

EFKKAVVSLIDEYFSNGDVDLAASDLRELGSSEYYPYFIKRLVSMAMDRHDKEKEMASVL

LSALYADVISPTQIRDGFFMLIESADDLAVDILDAVDILALFLARAVVDDILPPAFLARA

RKALPESSKGAQVVQTAEKSYLSAPHHAELVERRWGGSTHI-TVEEMKKKIADLLKEYVD

-SGETLEACRCIRELGVAFFHHEVVKKALVLAMEIP-SAEPLLLKLLKEAAAEGLISSSQ

MVKGFSRLEEGLDDLALDIPSAKALFQSFVPKAISEGWLDASFDNPAGEN----------

-------------------------------GEFQVE-DENVRKYKKEAVTIIHEYFLSD

DIPELIRSLEDL--GA-PEYNPIFLKRLITLALDRKNREKEMASVLLSALHIEIFSTEDI

VNGFVMLLENAEDTTLDILDASNELALFLARAVIDDVLAPLNLDEIGSR----LPPKCS-

GSETVRMARTLSSARHAGERLLRCWGGG------TGWAV-EDAKDKITKLLEEYESGGVV

GEACQCIRDLGMPFFNHEVVKKALVMAMEKK-----------------------------

-----------NDR-MLDLLQECFSEGLITTNQLTKGFTRIKEGLDDLALDI-PNAKEKF

AFYVEHAKTKGWL

>Glycine_max_XP_003525619

EFKKAVVSIIEEYFSNGDVELASSDLKELGSCEYYPYFIKRLVSVAMDRHDKEKEMASVL

LSALYADVISPAQIRDGFFILLESADDLAVDILDAVDILALFLARAVVDDILPPAFLARA

KKALPESSKGVQVIQTAEKSYLSAPHHAELVERRWGGSTHI-TVEEVKKKIADLLREYVD

-SGDTLEACRCIRELGVSFFHHEVVKRALILAMEIR-SAEPPMLKLLKEAAEEGLVSSSQ

MVKGFSRLAESLDDLALDIPSAKALFQSFVPKAISEGWLDASLTKPATED----------

-------------------------------GEIQ-E-DEKVRKYKKESVTIIHEYFLSD

DIPELIQSLEDL--GA-PEYNPIFLKKLITLAMDRKNREKEMASVLLSALHIEIFSTEDI

VNGFVMLLESAEDTALDILDASNELALFLARAVIDDVLAPLNLEEIGCR----LPPKCS-

GSETVRMARSLIAARHAGERLLRCWGGG------TGWAV-EDAKDKIMKLLEEYESGGVV

SEACQCIRDLGMPFFNHEVVKKALIMAMEKK-----------------------------

-----------NDR-MLDLLQECFSEGLITINQMTKGFTRIKDGLDDLALDI-PNAKEKF

GFYVEHAQSNGWL

>Gossypium_raimondii_Gorai001G0620001

EYKKAVVSIIEEYFSTSDVELAASDLKDLGSSEFHPYFIKRLVSIAMDRHDKEKEMASVL

LSSLYADVISPNQIRDGFVMLLDAADDLAVDILDAVNILALFVARAVVDEILPPAFLTRA

KKTLPESSKGYQVLQTAEKSYLSAPHHAELLERRWGGSIHV-TVEEMKKKIADLLREYVE

-SGDTFEACRCIRELGVSFFHHEVVKRALVLAMEIQ-AAELLMLKLLKEAAEEGLISSSQ

MVKGFARLAESLDDLALDIPSAKTLFQSIVPKAISEGWLDASFTKSSCED----------

-------------------------------GEGQSE-EKRLRRYKEEVVTIIHEYFLSD

DIPELIRSLEDL--GL-PEFNPIFLKKLVTLALDRKNREKEMASVLLSALHIEIFSTEDI

VNGFVMLLESAEDTALDILDASTELALFLARAVIDDVLAPLNLDEIASK----LSPNCS-

GSETIRMARSLITARHAGERLLRCWGGG------TGWAV-EDAKDKIMKLLEEYESGGVV

AEACQCIRDLGMPFFNHEVVKKALVMAMEKK-----------------------------

-----------NDR-MLNLLQVCFDEGLITINQMSKGFTRVKDGLDDLALDI-PNAKDKF

SFYMEYAQKKGWL

>Gossypium_raimondii_Gorai003G1088001

EYKKAVVSIIEEYFSTSDVEVAASDLKDLGSSEYHPYFIKRLVSMAMDRHDKEKEMASVL

LSSLYADVISPPQIRDGFVMLLESADDLAVDILDAVDILALFVSRAVVDEILPPAFIARA

KKTLPESSEGYQVLQTAEKSYLSAPHHAELLERRWGGSTHV-TVEEMKKKIADLLREYVE
-SGDTFEACRCIRELGVSFFHHEVVKRALVLAMEIQ-TAEPFMLKLLKEAAEEGLISSSQ
MVKGFARLAESLDDLALDIPSAKTLFQSIVPKAISQGWLDASFMKSSCTD----------
-------------------------------GEAQNE-DKKLDRYKKEIVTIIHEYFLSD
DIPELIRSLEDL--GL-PEYNPIFLKKLITLAMDRKNREKEMASVLLSALHIEIFSTEDI
GNGFVMLLESAEDTALDILDASNELALFLARAVIDDVLVPLNLEEITSK----LPPNCS-
GSETVRMARSLITARHAGERLLRCWGGG------TGWAV-EDAKDKIMKLLEEYESGGVV
AEACQCIRDLGMPFFNHEVVKKALVMAMEKK-----------------------------
-----------NDR-MLDLLQVCFNEGLITINQMTKGFTRVKDGLDDLALDF-PNAKDKF
SFYTEYAQKKGWL
>Theobroma_cacao_Thecc1EG014544t1
EYKKAVVSIIEEYFSTSDVELAASDLKDLGSSEYHPYFIKRLVSMAMDRHDKEKEMASVL
LSALYADVISPAQIRDGFVMLLESADDLAVDILDAVDILALFIARAVVDEILPPAFLTRA
KKTLPESSKGYQVLQTAEKSYLSAPHHAELLERRWGGSTHV-TVEEVKKKIADLLREYVE
-SGDTFEACRCIRELGVSFFHHEVVKRALVLAMEIQ-AAEPLMLKLLKEAAEEGLISSSQ
MVKGFARLAESLDDLALDIPSAKTLFQSIVPKALSEGWLDASFMKSSYED----------
-------------------------------GEAQNE-DKKLRQYKEEVVTIIHEYFLSD
DIPELIRSLEDL--GL-PEFNPIFLKKLITLAMDRKNREKEMASVLLSALHIEIFSTEDI
VNGFVMLLESAEDTALDILDASNELALFLARAVIDDVLVPLNLEDIASK----LPSNCS-
GSETVRMARSLIAARHAGERLLRCWGGG------TGWAV-EDAKDKIMKLLEEYESGGVV
AEACQCIRDLGMPFFNHEVVKKALVMAMEKK-----------------------------
-----------NDR-MLDLLQECFNEGLITINQMTKGFTRVKDGLDDLALDI-PNAKDKF
SFYIEYAQKKAWL
>Vitis_vinifera_XP_002277813
EYKKAVVSIIEEYFSTGDVELAASDLRELGSNEYHPYFIKRLVSMAMDRHDKEKEMASVL
LSALYADVISSAQISQGFFILLESADDLAVDILDAVDVLALFIARAVVDDILPPAFLTRA
KKTLPESSKGHQVIQTAEKSYLSAPHHAELVERRWGGSTHI-TVEEVKKKIADLLREYVE
-SGDAFEACRCIRELGVSFFHHEVVKRALVLAMEIR-TAEPLILKLLKEAAEEGLISSSQ
MLKGFARLAESLDDLALDIPSAKTLFELLVPKAISQGWLDASFLKPAGED----------
-------------------------------GEVHNEDDEKVRRFKEEAVAIIHEYFLSD

DIPELIRSLEDL--GM-PKFNPIFLKKLITLAMDRKNREKEMASVLLSSLHIEIFSTEDI

VNGFVMLLESAEDTALDVLDASNELALFLARAVIDDVLAPLNLEEIGSK----LPPNCS-

GSETVHMARSLIAARHAGERILRCWGGG------TGWAV-EDAKDKIMKLLEEYESGGDV

GEACQCIRDLGMPFFNHEVVKKALVMAMEKK-----------------------------

-----------NDR-MLDLLQECFCEGLITINQMTKGFGRIKDGLDDLALDI-PNAEEKF

SFYVEYARKMGWL

>Populus_trichocarpa_XP_002318100

DYKKAVVSIIEEYFSTGDVEVAASDLRELGSSEYHLYFIKRLVSMAMDRHDKEKEMASVL

LSALYADVISPSQIRDGFVILLESADDLAVDILDAVDILALFIARAVVDDILPPAFLTRA

KKTLPESSKGFQVLQTAEKSYLSAPHHAELVERKWGGSTHI-TVEEVKKKIADLLREYVE

-SGDAVEACRCIRELGVSFFHHEVVKRALVLAMEIR-TAEPLILKLLKEASEEGLISSSQ

MAKGFARLTESLDDLALDIPSAKSLFQSLIPKAIAEGWLDASFMKSSGED----------

-------------------------------GQVQAE-YEKVKRFKEEVVTIIHEYFLSD

DIPELIRSLEDL--GM-PECNPIFLKKLITLAMDRKNREKEMASVLLSALHIEIFSTDDI

VNGFVMLLESAEDTALDILDASNELALFLARAVIDDVLAPLNLEEIGSK----LPPNCS-

GSETVRMARSLIAARHAGERLLRCWGGG------TGWAV-EDAKDKILKLLEEYESGGVV

GEACQCIRDLGMPFFNHEVVKKALVMAMEKK-----------------------------

-----------NDR-MLDLLQVCFNEGLITINQMTKGFNRIKDGMDDLALDI-PNAEEKF

SFYVEYAQKKGWL

>Ricinus_communis_XP_002511272

EYKKAVVSIIEEYFSTGDVEVAASDLRELGSSQYHPYFIKRLVSMAMDRHDKEKEMASVL

LSTLYADVIISSQIRDGFVILLESADDLAVDILDAVDILALFIARAVVDDILPPAFLTRA

KKTLPESSKGFQVLQTAEKSYLSAPHHAELVERRWGGSTHI-TVEEVKKKISDLLREYVE

-NGDAFEACRCIRELGVSFFHHEVVKRAIILAMEIR-TAEPLILKLFKEASEEGLISSSQ

MVKGFARLAESLDDLALDIPSAKALFQSLVPKGISEGWLDASFMKSSSED----------

-------------------------------GLGQAE-DKRLRGYKEEIVTIIHEYFLSD

DIPELIRSLEDL--GM-PEFNPIFLKKLITLAMDRKNREKEMASVLLSALHIEIFSTEDI

VNGFVMLLESAEDTALDILDASNELALFLARAVIDDVLAPLNLEEIGSK----LPPNCS-

GTETVYMARSLIAARHAGERILRCWGGG------TGWAV-EDAKDKIMKLLEEYESGGVV

NEACQCIRDLGMPFFNHEVVKKALVMAMEKK-----------------------------

-----------NDR-MLDLLQACFDEGLITINQMTKGFTRIKDGLDDLALDI-PNAKEKF

SFYVEYAQRKGWL

>Physcomitrella_patens_XP_001777050

EYKEKVVSLIEEYFASGDVLSMATDLSNLGSPNFHHHFVKKLISMAMDHHDKEKEMASVL

LSALYADVIQPDQLAKGFTNLLESVDDLILDIPEAVDILAIFLARAVVDDILPPAFLSKT

MKVLAEGSQGLAVIQKTEKSYLSAPHHAEVIERKWGGSTHT-TVAEVQAKIVALLKEYVE

-SGDNAEACRCIRELNVPFFHHEVVKKALVLAMEEP-AAEGKIWSLLKEAAEEGLITSSQ

MSKGFTRISDSIHDLALDIPQAKEKLETFTTKAVEEGWVSAPFSRAVVSE----------

-------------------------------LMAGSAESQEARAFKAQATNIIQEYFLSA

DINEVVTSLEDL--AA-PDYHAAFVKRLITLAMDRKYREKEMASILVSELYAEVISIASI

ARAYTLLLQSAEDTALDIPDAANELSLFLARAVIDDILAPLYLEEISEQ----LAEGSL-

GREIVRMAQSVLSARHAGERILRCWGGG------GTGEALEDAKDKIKSLLEEFEAGGEL

GEACQCIRDLDMSFFHHEVVKKAVVMAIEKN-----------------------------

-----------NPR-LLMLLQECANEGLITTSQMTKGFSRVMDALDDLSLDN-PGAKDKA

AQYVEQAKKEGWL

>Physcomitrella_patens_XP_001754785

EYKGKVVSLIEEYFAAGDVASMATDLSNLESPNYHHHFVKKLISMAMDHHDKEKEMASVL

LSALYADVLKPEQLAKGFTNLLESVEDLVLDIPEAVDILAIFLARAVVDDILPPAFLSKT

RKLLVDGSQGLVVVQKAEKSYLSAPHHAEIIERKWGGSTHT-TVAEVQAKIVTLLKEYVE

-SGDKAEACRCIRELNVPFFHHEVVKKALVLAMEEP-AAEGKLWSLLIETAEEGLITSSQ

MSKGFTRISDSIHDLALDIPQAKDKLESFTSKAVEEGWVSAPFSRAVVSE----------

-------------------------------LGAGTVGIQEARAFKANATNIIQEYFLSS

DISEVITSLEDL--AA-PDYHAAFVKRLILLALDRKNREKEMASVLVSELYAEVISIASI

ARAYTLLLQSAEDTSLDIPDAANQLSLFLARAVVDDILAPLHLDEISEQ----LVEGSL-

GREIVRMAQSMLSARHAGERILP----------------LEDAKEKIKSLLEEFDAGGEL

SEACQCIRDLDMSFFHHEVVKKAVVMAIEKN-----------------------------

-----------SSR-PLTLLKECANEGLITTSQMTKGFSRVMDALDDLALDN-PDAKDKA

AQYVEQAKKEGWL

>Amborella_trichopoda_URDJ-37850

DYKKVVVVIIEEYFSTCDVETAASDLRELAASDYHHYFVK-----AMDRHDREKEMASVL

LSALYADVISPEQIRQGFALLLDSADDLAVDIPD-----ALFVARAVVDDIVPPAFLTRA
KKTLPEASKGLEAVQAAEKSYLSAPHHA-----EFGGTTHI-TVEEAKKKIGDILSEYVE
-SGDTGEACRCIRELGLPFFHHEVVKRME-----KP-ASRGLILSLLREAAEEGLISSSQ
MSKGFGRLAESLDDLSLDVPTAR-----LAPKAVSEGWLDPSFPKASTMA----------
-------------------------------GELEE--DEELRRFKEEVVAIIHEYFLSD
D-----RSLEDL--NA-PKYNPVFLKKLITLAMDRKNHEKEMASVLLSALYTDVFSAED-
----VMLLESADDISIDILDASNELAMFLARAVIDDTLVPLNLEEIASK----LPP----
--NCVHMAKSLLSARHAGERILRCWGGG------TGWAV-EDAKDKITKLLEEFESGGDV
G-----IRELGMPFFNHEVVKKALVMAMEKK-----------------------------
-----------NDR-MLDLLQECFGEGLITINQMTKG-----DSLDDLALDI-PDAREKF
KVYYEHARQSAWI
>Austrobaileya_scandens_FZJL-1758
DYKKALVAIIEEYFSTDDVELVASDLRDLSSQEYHHYFVK-----KLDRHDKEKEMASVL
LSALYADVISSDQISQGFMMLLEAADDLALDIPN-----TVEIARAVVDDILPPAFLTRA
KEKFSESSKGLQVILTAEKSYLSAPHHA-----EFVETTHI-TVEEVKKKIADLLREYVE
-SGDTAEACRCIRELGVSFFHHEVVKRAL-----IR-SAELLILKLLKEAATEGLISSSQ
MIKGFGRLAESLDDLSLDIPSAK-----PMPKAISEGWLNASFLKSLGPA----------
-------------------------------GELRDNEDDNVRQFKEEAVTIIHEYFLSD
D-----RSLEDL--AA-PEFNPVFLKKLITLAMDRKNREKEMASVLLSALHTEIFSTED-
----VMLLESAEDTALDILDASNELALFLARAVIDDILVPLNLEEISSN----LPP----
--NCVHMARSLLTARHAGERILRCWGGG------TGWAV-EDAKDKIAKLLEEYESGGVV
G-----IRDLGMPFFNHEVVKKALVMAMEKK-----------------------------
-----------NDR-MLDLLQECFNEGLITINQMTKG-----DSLEDLALDI-PNAEEKF
GFYVDHARKNGWL
>Austrobaileya_scandens_FZJL-1759
DYKKALVAIIEEYFSTDDVELVASDLRDLSSQEYHHYFVK-----KLDRHDKEKEMASVL
LSALYADVISSDQISQGFMMLLEAADDLALDIPN-----TVEIARAVVDDILPPAFLTRA
KEKFSESSKGLQVILTAEKSYLSAPHHA-----EFVETTHI-TVEEVKKKIADLLREYVE
-SGDTAEACRCIRELGVSFFHHEVVKRAL-----IR-SAELLILKLLKEAATEGLISSSQ
MIKGFGRLAESLDDLSLDIPSAK-----PMPKAISEGWLNASFLKSLGPA----------

-------------------------------GELRDNEDDNVRQFKEEAVTIIHEYFLSD
D-----RSLEDL--AA-PEFNPVFLKKLITLAMDRKNREKEMASVLLSALHTEIFSTED-
----VMLLESAEDTALDILDASNELALFLARAVIDDILVPLNLEEISSN----LPP----
--NCVHMARSLLTARHAGERILRCWGGG------TGWAV-EDAKDKIAKLLEEYESGGVV
G-----IRDLGMPFFNHEVVKKALVMAMEKK-----------------------------
-----------NDR-MLDLLQECFNEGLITINQMTKG-----DSLEDLALDI-PNAEEKF
GFYVNHARKNGWL
>Selaginella_moellendorffii_XP_002967718
VYKEKVVYIVEEYFATGNVADAATDLRDLGSPSYHHYFVKKLISMALDRHDREKEMASVL
LSALYADVMEPDQLAKGFTKLLESADDLALDIPDATDVLALFLARAVVDDILPPAFLSKT
QAVLPDGSKGLEVLQNAEKSYLNAPLHAEVVERRWGGSTQT-TVEEVKRKITDLLKEYVE
-SGDQAEARRCIRELNVPFFHHEVVKKALTLVIEKK-SAEAGIVSLLQECADEGLISSNQ
MSKGFSRVFDALEDLSLDVPQAREILEDLAAKAMSAGWLSSSFVTPAAASAAAAA-----
----------------------AAASNDDGAAANGDTAVDDAHVFKQKATGIIQEYFLSD
DIAEVVRSLEDL--AA-PDFNAVFVKKLITLAMDRKNREKEMASVLLSALYAEVIPVGQI
GKAFTMLLESAEDTALDIPDAANQLAYFLDRAVVDDILPPLYLEEINEQ----LPEGSL-
GKEIVHSAESILAARHAGERILRCWGGG------TGRAVLDDAKDKIAKLLEEFESGGDM
GEACQCVRDLAMPYFHHEVVKRALVMAMERK-----------------------------
-----------SAR-PLCFLRECSSEGLITTSQMVKGFGRVADYIDDLALDI-PDAKSKF
QGFVDEAKAQGWL
>Picea_abies_MA_10693g0010
EYKRKVGSIIKEYFATDDVASVAADLADLGCPSYHHYFVKKLISMAMDRHDREKEMAAVL
LSSLYADVIEPEQVSK------------------------------VVDDILPPAFLTKT
SKSLPEDSKGVEVIQKAEKSYLSAPLHAEIIERRWGGSTHV-TVEEVKKKISDLLNEYVQ
-SGDKAEACRCIRDLKVRFFHHEVVKRALILAMERR-SAEPLILALLKEAAEEGLITSSQ
MSKGFGRLADTIDDLSLDIINAKDMFESLIFKATTEGWLSPASVSS--------------

>Picea_abies_MA_2314g0010
EYKGKVGSIIEEYFATDDVAAAAADLADIGCPSYHHYFVKKLISMAMDRHDREKEMAAVL
LSSLYADVIEPEQVSKGFSKLLDTADDLALDIPEAVDILALFVARAVVDDILPPAFLTKT
LKSLPEDSKGVEVIQKAEKSYLSAPLHAEIIERRWGGSTHI-TVEEVKRKISDLLNEYVE
-SGDKAEACRCIRDLNVPFFYHEVVKRALILAMERR-SAEPLILVLLKEAAEEGLITSSQ
MSKGFGRLADTVDDLSLDIINAKDMFESLVLKATTEGWLSPACVKSIP------------
------------------------------SQRNGPAEHDNARLFKERAVTIIQEYFLSD
DIFEVVRSLEDL--AA-PDFNAVFVKRLITLAMDRKNREKEMASVLLSALYTEVFPVEDI
VNGFVLLLESAEDTALDIPFAANELALFLARAVIDDVLAPLNLEEIDNQ----LNPGSI-
GSEIVHMARSLLAARLAGERILRCWGGG------TGWAV-EDAKDKINKLLEEYETGGDI
GEACQCIRDLNMKFFHHEVVKKALVMAMEKK-----------------------------
-----------NDW-LLNLLQECSSEGLITINQMMKGFTRVADSLDDLALDI-PNAREKF
ASYVEQAKMNGWL
>Sundacarpus_amarus_13902
EYKGKVGSIIEEYFATDDVAAAAADLSDLGCPNYHHYFS-----MAMDRHDREKEMAAVL
LSSLYADVIEPEQVSKGFSKLLESADDLALDIPL-----ALFVARAVVDDILPPAFLTKT
LKSLTEDSKGVEVIQKAEKSYLSAPLHA-----RWGGCTHI-TVEEMKKKISDLLNEYVE
-SGDKVEACRCIRDLSVPFFHHEVV-----LAMEKR-NAEPFILDLLKEAAEECLITSSQ
MSKGFGRLADTVDDLSLDILNQ-----SLILKATTEGWLSPESVRSIP------------
------------------------------SQQNGPGEEDNVRLFKERSGTIIKEYFL--
---EVVRSLEDL--AA-PDFNALFIKKLITLAMDRKNREKEMASVLLSALYTEVFPQ---
--GFTLLLDSAEDTALDIPDASNELALFLARAVVDDILAPLNLEEISSQ----MK-----
-SEIVRMVRSLLSARLAGERILRCWGGG------TGWAV-EDAKDKINKLLEEYEAGG--
---CQCIRDLNMPFFHHEVVKKALVMAMEKK-----------------------------
-----------NDR-VLNLLQESSSEGLITINQMR-----VADSLDDLALDL-PNAKRKF
ASYVEQAKQNGWL
>Cycas_micholitzii_XZUY-1711

EYKVKVGSIIEEYFATDDVGSTAADLSDLGCPSYHHYFV-----MAMDRHDREKEMAAVL

LSALYADVIEPEQVSKGFNKLLESADDLALDIPD-----ALFVARAVVDDILPPAFLTKT

LKALPEDSKGIEVIQKAEKSYLSAPLHA-----EWGGSTHN-TVEEVKKKMSDLLNEYVE

-SGDKAEACRCLRDLNVPFFHHEVV-----KAMERR-SAESLLLALLKEAAEEGLITSSQ

MSKGFGRLADTVDDLSLDILNA-----SLIFKATAEGWLSPSSVKSIP-----------

-----------------------------TQLNGPVEHDNARVFKEKAVTIIQEYFL--

---SVVRSLEDL--AA-PDFNAVFVKKLITLAMDRKNREKEMASVLLSALYTEVIPV---

--GFVLLLEDAEDTALDIPDAANELALFLARAVIDDVLAPLNLEEISSQ----LR-----

-PEIVHMARCLLAARLAGERILRCWGGG------TGWAV-EDAKDKINKLLEEYETGG--

---DQCIRDLDMPFFHHEVVKKALVMAMEKK----------------------------

-----------NDR-LLNLLQECSSEGLITINQMM-----VADSLDDLALDI-PNAKEKF

TSYVEQAKDGGWL

>Illicium_floridanum_VZCI11530

NYKKAVVPIIEEYFSTGDVELVASDLRDLSCQEYHPYFVK-----RLDRHDKEKEMASVL

LSALYADLISSPQISQGFMMLLESSDDLALDILD-----TADIARAVVDDILPPAFVTKA

KKKFSESSKGFEVLSTAEKSYLSAPHHA-----EFVETTHV-TVEEIKKKMADLLREYVE

-SGDTAEACRCIRELGVPFFHHEVVKRSL-----IR-SAEPLILKLLKEAATEGLISSSQ

MIKGFGRLAESLDDLSLDIPTAE-----SMEKAISEMWLDASILKCSAPA----------

-----------------------------GELRENGNGDDCASRFKEEAVTIIHEYFLSL

-----IRSLEDL--AA-PEFNPIFLKKLITLAMDRKNREKEMTSVLLSAIHTDIFTT---

--GFVMLLESAEDTALDILDASNELALFLARAVIDDVLVPLNLEEISSK----LPP----

--ETVHMARSLLAARHAGERILRCWGGG------TGWAV-EDAKDKIAKLLEEYESGG--

---DQCIRDLGMPFFNHEVVKKALVMAMEKQ----------------------------

-----------NDR-MLDLLEESFSEGLITINQMTV-----RDSLKDLSLDI-PNAEEKF

VLYVEHARKNDWL
